# Characterization of the non-glandular gastric region microbiota in *Helicobacter suis*-infected versus non-infected pigs identifies a potential role for *Fusobacterium gastrosuis* in gastric ulceration

**DOI:** 10.1186/s13567-019-0656-9

**Published:** 2019-05-24

**Authors:** Chloë De Witte, Kristel Demeyere, Sofie De Bruyckere, Bernard Taminiau, Georges Daube, Richard Ducatelle, Evelyne Meyer, Freddy Haesebrouck

**Affiliations:** 1 Department of Pathology, Bacteriology and Avian Diseases, Faculty of Veterinary Medicine, Ghent University, Merelbeke, Belgium; 2 Department of Pharmacology, Toxicology and Biochemistry, Faculty of Veterinary Medicine, Ghent University, Merelbeke, Belgium; 3 Department of Food Sciences, FARAH, University of Liège, Liège, Belgium

## Abstract

*Helicobacter suis* has been associated with development of gastric ulcers in the non-glandular part of the porcine stomach, possibly by affecting gastric acid secretion and altering the gastric microbiota. *Fusobacterium gastrosuis* is highly abundant in the gastric microbiota of *H. suis*-infected pigs and it was hypothesized that this micro-organism could play a role in the development of gastric ulceration. The aim of this study was to obtain further insights in the influence of a naturally acquired *H. suis* infection on the microbiota of the non-glandular part of the porcine stomach and in the pathogenic potential of *F. gastrosuis*. Infection with *H. suis* influenced the relative abundance of several taxa at phylum, family, genus and species level. *H. suis*-infected pigs showed a significantly higher colonization rate of *F. gastrosuis* in the non-glandular gastric region compared to non-infected pigs. In vitro, viable *F. gastrosuis* strains as well as their lysate induced death of both gastric and oesophageal epithelial cell lines. These gastric cell death inducing bacterial components were heat-labile. Genomic analysis revealed that genes are present in the *F. gastrosuis* genome with sequence similarity to genes described in other *Fusobacterium* spp. that encode factors involved in adhesion, invasion and induction of cell death as well as in immune evasion. We hypothesize that, in a gastric environment altered by *H. suis*, colonization and invasion of the non-glandular porcine stomach region and production of epithelial cell death inducing metabolites by *F. gastrosuis*, play a role in gastric ulceration.

**Electronic supplementary material:**

The online version of this article (10.1186/s13567-019-0656-9) contains supplementary material, which is available to authorized users.

## Introduction

Gastric ulceration is a common disease entity of pigs worldwide, with prevalences of up to 93%. It may result in decreased daily weight gain, decreased feed intake and sudden death, leading to significant economic losses and animal welfare issues. The aetiology is multifactorial. Diet particle size, management and infection with pathogens are factors that have been hypothesized to be involved [1]. The exact pathophysiological mechanism behind porcine gastric ulceration, however, is not completely clear. In marked contrast with human patients and several other animal species, gastric ulcers do not develop in the glandular part of the porcine stomach, but are almost exclusively found in the *Pars oesophagea*, a small area around the opening of the oesophagus which does not contain glands. Since this stomach region is not protected by mucus, it is highly susceptible to irritation by hydrochloric acid produced in the distal gland zone of the porcine stomach. Chronic irritation of the *Pars oesophagea* results in hyperkeratosis, erosion and finally ulceration [1, 2].

*Helicobacter suis* is a zoonotic bacterium that colonizes the gastric mucosa of pigs worldwide. Results of recent studies indicate that *H. suis* infection plays a role in porcine gastric ulcer disease, probably by affecting gastric acid secretion through alteration of the number and/or function of parietal, D- and G-cells [3, 4]. Impaired gastric acid secretion, induced by *H. suis*, may favor the establishment of specific gastric microbiota. Our metagenomics study of pooled samples from the different stomach regions (i.e. *Pars oesophagea*, cardiac, fundic and pyloric gland zones) from *H. suis*-positive and -negative 6–8 months old pigs revealed that a novel *Fusobacterium* sp., designated *F. gastrosuis*, was highly abundant in the gastric microbial community of *H. suis*-infected animals [5].

Fusobacteria are normal constituents of the oropharyngeal, gastrointestinal and genital microbiota of a wide range of animal species, going from mammals to birds and fish. Nevertheless, they are also frequently isolated from clinical samples of both human and animal origin, especially from cases of pyonecrotic infections [6]. Infections with *Fusobacterium* spp. have been linked to a wide range of pathologies. Furthermore, genome analysis of *Fusobacterium* spp. showed the presence of a large and diverse set of virulence associated genes, such as leukotoxin and immunosuppressive protein A (fipA), which have been associated with cell death and immuno-evasion, respectively [7]. Additionally, some *Fusobacterium* spp. were able to adhere to and actively invade host cells without the aid of other factors, due to the presence of genes encoding adhesins and membrane-related proteins, while others only aggravated necrosis when tissue damage was initiated by other microorganisms or environmental factors [6].

We hypothesize that, in a gastric environment altered by *H. suis*, colonization and invasion of the *Pars oesophagea* and production of epithelial cell death inducing metabolites by *F. gastrosuis* may play a role in gastric ulceration.

The overall aim of the current study was to obtain further insights in the influence of a naturally acquired *H. suis* infection on the microbiota of the *Pars oesophagea* of the porcine stomach and in the pathogenic potential of *F. gastrosuis* upon co-infection. Therefore, the ability of *F. gastrosuis* to induce cell death in oesophageal and gastric epithelial cell lines was first determined and its genome was also analysed for the presence of genes encoding putative virulence factors which may be involved in adhesion, invasion and host cell death as well as in immuno-evasion. Secondly, the colonization rate of *F. gastrosuis* and its abundance in the microbiota of the *Pars oesophagea* was compared in *H. suis*-infected and non-infected pigs.

## Materials and methods

### Study 1: Microbiota composition of the *Pars oesophagea*

#### Sampling of porcine stomachs

Ten *H. suis*-positive and 10 *H. suis*-negative stomachs of 6–8 months old pigs used in another study from our group were further analysed [8]. Using autoclaved tweezers and scalpels, a biopsy of 40–50 mg consisting of mucosa and submucosa was taken from the *Pars oesophagea* for gastric microbiota analysis and subsequent *F. gastrosuis* quantification by quantitative real time (RT)-PCR was realized. In addition, a second biopsy consisting of mucosa and submucosa was taken from the *Pars oesophagea* to determine mRNA expression levels of genes encoding host factors (markers) involved in inflammation, hyperkeratosis and ulceration.

#### Gastric microbiota analysis

DNA was extracted from the gastric biopsy of the *Pars oesophagea* using the DNeasy Blood & Tissue Kit (Qiagen, Hilden, Germany) according to the instructions of the manufacturer. *16S rRNA* gene amplicon pyrosequencing was performed using the Roche GS-Junior Genome Sequencer as described previously [9]. The obtained 16S rDNA sequence reads were processed using MOTHUR (software package v1.35), Pyronoise algorithm and UCHIME algorithm for alignment and clustering, denoising and chimera detection, respectively [10, 11]. The obtained read sets were compared to a reference dataset of aligned sequences of the corresponding region derived from the SILVA database (v1.15) of full-length rRNA sequences implemented in MOTHUR [12]. The final reads were clustered into operational taxonomic units (OTUs) using the nearest neighbor algorithm using MOTHUR with a 0.03 distance unit cut-off. A taxonomic identity was attributed to each OTU by comparison with the SILVA database using a 80% homogeneity cut-off. When a taxonomic identification lower than 80% was obtained, the taxonomic level was labelled with the first defined level from higher level followed by the label “unclassified”. Finally, the unique sequences for each OTU were compared with the SILVA data set using the BLASTN algorithm. For each OTU, a consensus taxonomic identification was given when less than 1% of mismatch with the aligned sequence was obtained. In the final metadata table, the following labelling was used: the population is identical to a taxonomically defined species and is labelled “genus_species”; the population is identical to a reference sequence belonging to a still undefined species and is labelled “genus_NCBI accession number”; the sequence is not identical to any known sequence and is labelled with the corresponding OTU number.

In order to determine the effect of *H. suis* on the gastric microbiota composition, the *H. suis*-negative and *H. suis*-positive groups were compared.

Subsampled datasets were obtained and evaluated in MOTHUR to estimate the richness, microbial diversity and population evenness by using the Chao1 estimator, Simpson’s reciprocal index and Simpson’s evenness index, respectively [13, 14]. Population structure and community membership were assessed with MOTHUR using distance matrix based on Bray–Curtis dissimilarity index. Differences in functional profiles of gastric bacterial communities were analysed by mapping taxa into several phenotypes (i.e. metabolism, Gram staining, sporulation…) using METAGENassist [15]. Only the phenotypes detected in more than 50% of the samples were included for further analysis.

#### Statistical analysis

Statistical differences in microbial diversity, richness and population evenness between the groups were investigated using non-parametric Kruskal–Wallis tests with Tukey post hoc tests using PRISM 7 (Graphpad Software). Using MOTHUR, community composition differences were investigated using Analysis of molecular variance (AMOVA) and homogeneity of molecular variance (HOMOVA). In order to highlight statistical differences in relative bacterial abundance between the groups, non-parametric Kruskal–Wallis tests with Tukey post hoc tests and Benjamini–Hochberg false discovery rate were performed using the STAMP software. Differences were considered statistically significant at a corrected *p*-value of less than 0.05.

### Study 2: *F. gastrosuis* colonization

#### Sampling of porcine stomachs

Sixty-eight stomachs of 6–8 months old pigs and 60 stomachs of adult sows (1–3 years old) were collected over a period of 10 months from two slaughterhouses in Flanders, Belgium. The pigs originated from different herds. Stomachs of 46, 2–3 months old pigs were collected from two different pig herds (23 samples from each herd). The stomachs of all pigs had been previously used [4]. Based on the method of Hessing [16], mucosal lesions of the *Pars oesophagea* were scored as follows: score 0 for normal mucosa, score 1 for mild hyperkeratosis encompassing less than 50% of the surface, score 2 for severe hyperkeratosis encompassing more than 50% of the surface, score 3 for hyperkeratosis with few erosions, score 4 for hyperkeratosis with several erosions and score 5 for hyperkeratosis with many erosions or ulceration. Using autoclaved tweezers and scalpels, biopsies of 40–50 mg consisting of mucosa and submucosa were taken from the *Pars oesophagea* as well as from the cardiac, fundic and pyloric gland zone for quantification of *F. gastrosuis* DNA by RT-PCR. In addition, a swab from the oral cavity was taken from each 2–3 months old pig using a MasterAmp™ Buccal Brush (Epicentre, Madison, USA), as well as biopsies of 40–50 mg consisting of mucosa and submucosa from jejunum and colon.

#### Quantification of *F. gastrosuis* by RT-PCR

DNA was extracted from the gastric biopsies from the *Pars oesophagea*, cardiac, fundic and pyloric gland zone from the different age groups and from the jejunum and colon biopsies from the 2–3 months old pigs using the DNeasy Blood & Tissue Kit (Qiagen, Hilden, Germany) according to the instructions of the manufacturer. In addition, DNA was extracted from the oral cavity swab using PrepMan Ultra Sample Preparation Reagent (Life Technologies, Carlsbad, California) according to the manufacturer’s instructions. The DNA extracted from the *Pars oesophagea* biopsies sampled for gastric microbiota analysis in study 1 was also included for *F. gastrosuis* quantification.

The presence of *F. gastrosuis* DNA was determined using a species-specific, RT-PCR based on the *gyrase B* (*gyrB*) gene [5]. The copy number of the obtained amplicons was calculated and converted to the number of *F. gastrosuis* bacteria per mg gastric tissue, by including tenfold dilutions of an external standard. For generating the standard, part of the *gyrB* gene (1212 bp) from *F. gastrosuis* strain CDW1 was amplified using UP-1 and UP-2r primers as previously described [5]. The standard consisted of tenfold dilutions, starting at 10^8^ PCR amplicons, for each 10 µL of reaction mixture. Two microliter of extracted DNA template was added to 10 µL reaction mixture, consisting of 0.25 µL of both primers (0.5 pmol/µL) located within the 1212 bp fragment (to yield a 158 bp PCR product), 3.5 µL HPLC water and 6 µL SensiMix™ SYBR No-ROX (Bioline Reagents Ltd, London, UK). Sense primer was GB2_F: 5′-GCA GCT CAA AGA GCA AGA GAA GCA-3′. Anti-sense primer was GB2_R: 5′-CTT CCC TGC TTT GCA GAA CCT CC-3′. The cycling conditions were initial denaturation at 95 °C for 10 min, followed by 47 cycles of 95 °C for 20 s, 60 °C for 30 s and 72 °C for 30 s after which the total fluorescence of the samples was measured. Both standards and samples were run in duplicate on a CFX384™ RT-PCR System with a C1000 Thermal Cycler (Bio-Rad, Hercules, California, USA).

#### Expression of markers for inflammation and ulceration

RNA was extracted from the gastric biopsies of the *Pars oesophagea* using the RNeasy Mini Kit (Qiagen, Hilden, Germany), according to the manufacturer’s instructions. The obtained RNA concentrations were measured using a NanoDrop spectrophotometer (Isogen Life Science, Utrecht, The Netherlands), after which the concentration of each sample was adjusted to 1 μg/µL, followed by cDNA synthesis using the iScript™ cDNA Synthesis Kit (Bio-Rad). Expression analysis was then performed for genes encoding host factors involved in inflammation [(interleukin (Il)-1β IL-6, IL-8, IL-10, CXCL13, interferon (IFN)-γ and tumor necrosis factor (TNF)-α] as well as in hyperkeratosis and ulceration [claudin 1, 2, 3, 4 and 18; keratin 6A; heat shock protein (Hsp) 27, 72 and 73; epidermal growth factor (EGF); basic fibroblast growth factor (BFGF); hepatocyte growth factor (HGF); transforming growth factor beta 1 (TGFB1); cyclooxygenase 2 (COX2); nitric oxide synthase 2 (NOX2); CXCL2; occludin; zonula occludens (ZO) 1 and 2]. The housekeeping genes HMBS, Cyc, RPL4 and HPRT1 were shown to have a stable mRNA expression and therefore included as reference genes [17, 18]. All primer sequences are shown in Additional file 1. The mRNA expression levels of the reference and target genes were quantified using a RT-PCR, as previously described [17]. No-template-control reaction mixtures were included and all samples were run in duplicate. The Ct-values were first normalized to the geometric mean of the Ct-values of the reference genes. Fold changes were calculated using ΔΔCt method with mean of Ct-values from the *H. suis*-negative group. Finally, for each target gene, the results were expressed as fold changes of the mRNA expression of the *H. suis*-positive group relative to mRNA expression level of the *H. suis*-negative group.

#### Statistical analysis

Statistical analysis was performed using SPSS statistics 24 (IBM, New York, USA). Differences in severity of *Pars oesophageal* lesions, number of colonizing *F. gastrosuis* bacteria and fold changes of the markers for inflammation, hyperkeratosis and ulceration were investigated using the non-parametric Kruskal–Wallis H test with Bonferroni correction. A *p*-value ≤ 0.05 was considered to be significant. Correlations between mucosal lesions, fold changes and the number of *F. gastrosuis* bacteria were examined using the Pearson correlation coefficient. Differences were considered statistically significant at *p* ≤ 0.05.

### Study 3: Effect of *F. gastrosuis* on epithelial neoplastic cell lines

#### Bacterial strains

*Fusobacterium gastrosuis* strains CDW1, CDW3, CDW6 and CDW8 were used for the in vitro cell experiments as previously described [5]. All strains were isolated from the *Pars oesophagea* of pigs suffering from gastric ulceration.

#### Cell lines and culture conditions

All in vitro cell experiments were performed with MKN7 (human gastric tubular adenocarcinoma cell line, Riken Cell Bank, Japan) and KYSE-450 (human oesophageal squamous cell carcinoma, ACC 387; Deutsche Sammlung von Mikroorganismen und Zellkulturen) cells. The MKN7 cells were cultured in 89% RPMI medium 1640 (supplemented with 1 mM l-glutamine, Invitrogen, Waltham, MA, USA), 10% fetal calf serum (FCS; HyClone, Logan, Utah, USA) and 1% penicillin–streptomycin (10 000 U/mL, Invitrogen). The KYSE-450 cells were cultured in 44.5% RPMI medium 1640 (supplemented with 1 mM l-glutamine, Invitrogen), 44.5% Ham’s F12 medium (supplemented with 1 mM l-glutamine, Invitrogen), 10% FCS (HyClone) and 1% penicillin–streptomycin (10 000 U/mL, Invitrogen). Penicillin–streptomycin was not added to the media for in vitro cell experiments with viable *F. gastrosuis* bacteria. The cells were seeded in plastic tissue culture flasks (VWR, Radnor, Pennsylvania, USA), maintained in a humidified incubator at 37 °C under 5% CO_2_ and passaged at least twice a week using 1% trypsin solution consisting of 88% trypsin diluent (8 g NaCl, 0.2 g KCl, 0.12 g KH_2_PO_4_, 0.91 g Na_2_HPO_4_ and 4 mL 0.5% phenol red solution/1000 mL aqua dest), 10% trypsin stock solution (Invitrogen) and 2% EDTA (2 g/100 mL trypsin diluent).

#### Cell-death inducing effects of *F. gastrosuis* lysates

Both MKN7 and KYSE-450 cell lines were incubated with *F. gastrosuis* lysates from strains CDW1, CDW3, CDW6 and CDW8 to determine cytotoxicity. To prepare the *F. gastrosuis* lysate, bacteria were incubated anaerobically for 2 days on Columbia blood agar plates (Oxoid, Basingstoke, United Kingdom). After incubation, they were harvested by centrifugation (368* g*, 10 min, 4 °C), washed twice with Hank’s Balanced Salt Solution without calcium and magnesium (HBSS−) (Invitrogen) and resuspended in HBSS− until an optical density (OD) of 2.9 at 660 nm was obtained (i.e. approximately 1 × 10^8^ bacteria/mL). The bacterial suspension was sonicated 20 times for 30 s and centrifuged (15 000* g*; 5 min, 4 °C) to remove cellular debris. The supernatant was then filtered through a 0.22 µm pore filter (Schleicher and Schuell, Munich, Germany) and stored at −80 °C. The resulting protein concentration was determined with the Pierce BCA protein assay kit (ThermoFisher Scientific, Waltham, Massachusetts, USA), according to the manufacturer’s instructions. The MKN7 and KYSE-450 cell lines were seeded at a concentration of 8 × 10^4^ cells/mL and 1 × 10^5^ cells/mL, respectively, in 6-well flat-bottom cell-culture plates (Greiner Bio One, Frickenhausen, Germany) and incubated at 37 °C and 5% CO_2_ until a monolayer was obtained after 48 h. Each well was washed twice with Hank’s Balanced Salt Solution with calcium and magnesium (HBSS+) to remove dead cells caused by the trypsin treatment and/or seeding. As previously described, each *F. gastrosuis* lysate was then added in different concentrations [50, 200 and 500 μg/mL, corresponding to a calculated theoretical multiplicity of infection (MOI) of approximately 5, 20 and 50 respectively] and incubated for 0, 24, 36 and 48 h [19]. As a positive control, *F. necrophorum* subps. *necrophorum* lysate (50, 200 and 500 μg/mL) was used. Untreated cells of both cell lines were included as negative controls, as well as positive controls for necrosis and apoptosis using 0.1% Triton X-100 and 1 μM staurosporine, respectively. Additional controls were used to quantify and compensate for spectral bleed-through. All conditions were performed in triplicate. After incubation, the cells were trypsinized and washed 3 times with HBSS− to remove cellular debris. Next, the cells were stained incubated for 10 min in the dark at room temperature with a labelling solution (10 mM 4-(2-hydroxyethyl)-1-piperazine ethanesulfonic acid (HEPES), 140 mM NaCl and 5 mM CaCl_2_) containing Annexin-V-fluorescein isothiocyanate (1/50 diluted Annexin-V-FITC; 11828681001, Sigma-Aldrich) and propidium iodide (1 µg/mL PI; P4864, Sigma-Aldrich). Acquisition and analysis was performed on a CytoFLEX flow cytometer using the CytExpert 2.0 software (Beckman Coulter, Indianapolis, Indiana, USA). Following terms were used to describe the different subpopulations: viable cells (Annexin-V-FITC negative, PI negative), early apoptotic cells (Annexin-V-FITC positive, PI negative) and late apoptotic/necrotic cells (Annexin-V-FITC positive, PI positive) [20]. A laser line of 488 nm was used with emission filters 525/40 and 585/42 for detection of annexin and propidium iodide, respectively. Before each measurement, calibration was performed to exclude instrument-related fluorescence intensity changes over time using CytoFLEX daily QC fluorospheres (Beckman Coulter). A minimum number of 10 000 events in the single, viable cell gate was analysed for each sample. The gating strategy for both cell lines is presented in Additional files 2, 3.

Simultaneously, the presence of apoptosis and/or necrosis was verified using light microscopy. The cells were harvested and cytospins were prepared by centrifugation at 55 *g* for 5 min. Slides were air-dried, fixed in methanol and stained with Hemacolor (Merck, Darmstadt, Germany).

#### Cell-death inducing effect of viable *F. gastrosuis* bacteria

Prior to infection, the viability of *F. gastrosuis* grown and harvested as described above was determined. Approximately 5 × 10^8^ bacteria/mL cell medium were added to 6-well flat-bottom cell-culture plates and incubated for 0, 6, 12, 24 and 36 h at 37 °C and 5% CO_2_. After incubation, the colony forming units (cfu) were determined by serial dilution and plating on Columbia agar plates (Oxoid, Basingstoke, UK) supplemented with 5% defibrinated sheep blood (E&O laboratories, Bonnybridge, Scotland, UK). The plates were incubated anaerobically for 3 days at 37 °C.

Both MKN7 and KYSE-450 cell lines were incubated with viable *F. gastrosuis* bacteria from strains CDW1, CDW3, CDW6 and CDW8 to determine the effect on cell death. The bacteria were incubated and harvested until an OD of 2.9 was obtained, as described above. After obtaining a monoculture of MKN7 and KYSE-450 cell lines (± 5 × 10^5^ cells/mL), these were inoculated with the different *F. gastrosuis* strains at a MOI of 5, 20 and 50 (i.e. corresponding to approximately 50, 200 and 500 μg/mL lysate, respectively) and incubated for 0, 2, 6 and 12 h. The percentages of viable, apoptotic and necrotic cells were determined using the CytoFLEX as described above.

#### Properties of cell death inducing *F. gastrosuis* component(s)

To determine whether the cell death inducing component(s) were of protein nature, *F. gastrosuis* bacteria were pre-treated with pronase, trypsine or proteinase K (all at 1 mg/mL, 2 h, 37 °C) (Sigma-Aldrich) prior to incubation with MKN-7 and KYSE-450 cells. Bacteria were also pre-treated with paraformaldehyde (1%, 1 h, room temperature) to determine if cell death inducing agents were associated with the bacterial surface. Finally, *F. gastrosuis* bacteria and lysates were heat treated (100 °C, 10 min) to investigate their heat-lability. The percentages of viable, apoptotic and necrotic cells were flowcytometrically determined as described above. Negative as well as positive controls (i.e. untreated *F. gastrosuis* bacteria and lysates, respectively) were included. The viability of *F. gastrosuis* bacteria after protease, paraformaldehyde and heat treatment was verified by incubation on Columbia blood agar plates (37 °C, 48–72 h, anaerobic) after which bacterial growth was compared with that of untreated *F. gastrosuis* bacteria.

#### Activation of the pyroptotic pathway

Similarly as shown for *F. nucleatum* [21], activation of the pyroptotic pathway was investigated by determining caspase-1 and IL-1β expression using Western blot analysis.

Both MKN-7 and KYSE-450 cell lines were incubated either for 0, 24, 36 and 48 h with 50, 200 and 500 μg/mL *F. gastrosuis* lysates from strains CDW1, CDW3, CDW6, or with 5, 20 and 50 MOI *F. gastrosuis* bacteria from strains CDW1, CDW3, CDW6 for 0, 6, 12, 24 and 36 h as described. After incubation, total cell extracts were obtained using RIPA buffer supplemented with a protease inhibitor cocktail, as previously described [22]. Protein concentration was determined with the Pierce BCA protein assay kit, according to the manufacturer’s instructions (ThermoFisher). After the samples were boiled for 5 min at 95 °C, a total of 100 μg was suspended in 200 μL loading buffer (i.e. 24 mL of 0.5 M Tris pH 6.8, 20 mL of 10% SDS, 10 mL glycerol, 5 mL of 0.5% bromophenol blue and 48 mL aqua dest) and loaded on a 10% self-prepared denaturing polyacrylamide gel. Pageruler™ plus prestained protein ladder with bands from 10 to 250 kDa (ThermoFisher) was used for molecular mass determination. Separation under reducing conditions was followed by blotting on a 0.45 µm nitrocellulose membrane (Bio-Rad). After blocking in Tris-buffered saline with 0.1% Tween 20 (TBS-T) and 5% milk powder for 1 h, overnight incubation with the primary anti-IL1β (0.1 μg/mL; AF-201-NA, R&D systems, Minnesota, USA) and anti-caspase 1 (0.1 μg/mL; AB207802, Abcam, Cambridge, UK) in TBS-T with 5% milk powder was carried out. Membranes were washed with TBS-T and horseradish peroxidase-conjugated donkey-anti-goat-antibody, respectively goat-anti-rabbit-antibody (each at 1:5000 in TBS-T with 5% milk powder) were each applied for 1 h. The blots were washed and protein bands were immunodetected by enhanced chemiluminescence, using Supersignal West Dura Extended Duration Substrate (ThermoFisher). The recombinant human IL-1β/IL-1F2 protein (201-LB, R&D Systems) and recombinant human caspase-1 protein (AB39901, Abcam) served as positive controls for the western blot analysis. Untreated MKN-7 and KYSE-450 cells served as negative control.

#### Statistical analysis

Statistical analysis was performed using SPSS statistics 24 (IBM, New York, USA). Differences in the percentage of viable, early apoptotic and late apoptotic/necrotic cells and cellular debris were investigated using the non-parametric Kruskal–Wallis H test with Bonferroni correction. A *p*-value ≤ 0.05 was considered to be significant.

### Study 4: Potential virulence associated genes in *F. gastrosuis*

#### Bacterial strain

The proteome of the *F. gastrosuis* type strain CDW1 has been previously determined (LT607734.1) [5]. In the current study, its proteome was further investigated for presence of genes encoding putative virulence factors which may be involved in adhesion, invasion and host cell death as well as immunoevasion.

#### Essential genes

The database of essential genes (DEG) version 15.2 consists of 53,885 essential protein-coding genes and 786 essential non-coding sequences from 48 prokaryotes and 26 eukaryotes. BLASTp search was performed for the proteome of *F. gastrosuis* against bacterial proteins of DEG with cut-off parameters of 1E−05 e-value and bit score of 100.

#### Potential virulence associated genes

Screening for presence of virulence associated genes was performed using the VirulentPred tool. The proteome of *F. gastrosuis* was also blasted against Pfam domains associated with virulence using the Pfam search tool. In addition, the proteome of *F. gastrosuis* was blasted against protein sequences from the virulence factor database (VFDB) core dataset (setA) with default parameters. This database contains 1796 virulence factors and 2599 curated virulence factor-related genes obtained from 74 bacterial pathogens. The query sequences having hit with cut-off bit score > 100 were considered as potential virulence factors. Finally, data on virulence factors from other *Fusobacterium* spp. reported in the literature was collected [7, 23–25], as well as data on genes characteristic for active invasion [6]. Homology search of these proteins was performed to find close matches in *F. gastro*s*uis* using BLASTP. The query sequences having an E-value < 1e−6 and an identity > 30% over at least 100 amino acids were hypothesized to have a similar structure and function.

## Results

### Study 1: Microbiota composition of the *Pars oesophagea*

Sufficient sequencing reads were obtained from the *Pars oesophagea* of all pigs (*n* = 20). Pyrosequencing yielded between 7479 and 7500 reads per sample. A total of 284,815 final reads were attributed to 3837 species level OTUs. Chimeric sequences represented 5–10% of the total sequencing reads and were excluded from the analysis.

**Figure 1:**
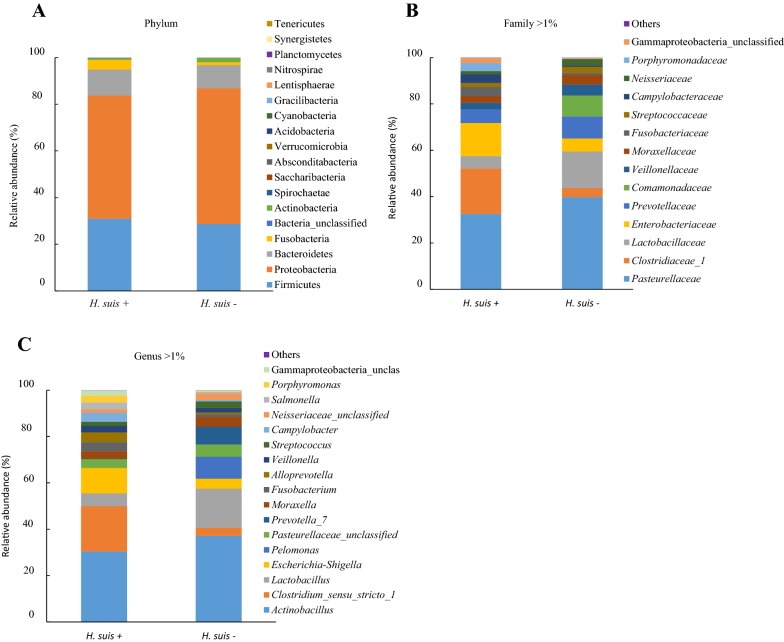
**Average bacterial community compositions in the** ***Pars oesophagea*** **of** ***H. suis*-positive and -negative pigs.** The cumulated histograms show the relative abundance of the identified taxa at phylum (**A**), family (**B**) and genus (**C**) level in the *Pars oesophagea* of *H. suis*-positive (*n* = 10) and -negative (*n* = 10) pigs. At family and genus level, taxa with a relative abundance < 1% are merged in the category “others”. The unclassified populations correspond to defined groups of the genus level for which a taxonomical classification assignation to the genus could not be attributed. These populations are therefore labelled with the first defined superior hierarchical taxonomic level followed by “_unclassified” to prevent confusion.

In general, total bacterial community analysis showed that the most dominant phylum in the porcine *Pars oesophagea* was Proteobacteria (56%), followed by Firmicutes (30%), Bacteroidetes (10%), Fusobacteria (3%) and Actinobacteria (1%). The relative abundance of other phyla was below 0.1%. On family level, following populations (i.e. > 1%) were dominant: *Pasteurellaceae* (33%), *Clostridiaceae*_1 (11%), *Lactobacillaceae* (10%), *Enterobacteriaceae* (9%), *Prevotellaceae* (7%), *Comamonadaceae* (4%), *Veillonellaceae* (3%), *Moraxellaceae* (3%), *Campylobacteraceae* (2%), *Fusobacteriaceae* (2%), *Neisseriaceae* (2%), *Porphyromonadaceae* (2%) and *Streptococcaceae* (2%). The major genera (i.e. > 1%) were *Actinobacillus* (29%), *Clostridium*_sensu_stricto_1 (10%), *Lactobacillus* (10%), *Escherichia* (7%), *Pasteurellaceae*_unclassified (4%), *Pelomonas* (4%), *Moraxella* (3%), *Prevotella*_7 (3%), *Alloprevotella* (2%), *Campylobacter* (2%), *Fusobacterium* (2%), *Neisseriaceae*_unclassified (2%), *Salmonella* (2%), *Streptococcus* (2%) and *Veillonella* (2%). The average gastric bacterial community composition at the phylum, family and genus level present in the *H. suis*-positive and -negative groups is represented in Figure 1, while the bacterial community composition of the *Pars oesophagea* of each individual pig is shown in Additional file 4.

Infection with *H. suis* had no effect on microbial diversity, richness and population evenness (Additional file 5). Furthermore, Unifrac-weighted analysis as well as AMOVA and HOMOVA did not reveal significant differences regarding community structure and composition of the groups. Population structure and community membership, as determined by Bray–Curtis dissimilarity index, were also not different between the groups.

In general, phenotypic analysis of the microbiota of the porcine *Pars oesophagea* revealed the presence of 19 metabolic phenotypes of which ammonia oxidizer, dehalogenation, nitrite reducer, sulfate reducer, sulfide oxidizer, chitin degradation, nitrogen fixation and xylan degrader were the most abundant, each accounting for 85%, 70%, 53%, 51%, 48%, 47%, 12% and 9% of the bacterial community, respectively. In the *Pars oesophageal* microbiota of all pigs, non-sporulating and Gram-negative bacteria were more abundant than sporulating and Gram-positive bacteria, respectively (38% vs 15% and 67% vs 26%, respectively). The *H. suis*-negative pigs showed a relative lower abundance of sporulating, nitrogen fixating and propionate metabolizing bacteria compared to the *H. suis*-positive pigs, although this difference was not significant (9% vs 21%, *p* = 0.075; 8% vs 17%, *p* = 0.063; 4% vs 12%, *p* = 0.064; respectively). Furthermore, *H. suis*-negative pigs showed a relative higher abundance of autotrophic bacteria compared to *H. suis*-positive pigs, which was borderline significant (68% vs 44%, *p* = 0.052).

Infection with *H. suis* influenced the relative abundance of several taxa at phylum, family, genus and species level, either significantly or borderline significantly. A detailed overview is presented in Table 1. In general, *H. suis*-positive pigs showed a relative higher abundance of the following taxa with at phylum level more Fusobacteria, at family level more *Fusobacteriaceae*, *Porphyromonadaceae*, *Bacteroidaceae*, *Clostridiaceae*_1, and at genus level more *Fusobacterium*, *Porphyromonas*, *Porphyromonadaceae_*unclassified, *Bacteroides*, *Alloprevotella*, *Clostridium_sensu_stricto_1*, *Leuconostoc*, *Pasteurella* and *Gammaproteobacteria*_unclassified. A relative lower abundance was detected for the following taxa with at family level less *Actinobacteria*_unclassified, *Bifidobacteriaceae*, *Corynebacteriaceae*, *Coriobacteriaceae*, *Streptococcaceae* and Gammaproteobacteriaceae_unclassified, at genus level less *Actinobacteria*_unclassified, *Bifidobacterium*, Corynebacteriaceae_unclassified, Lachnospiraceae_UCG-007, *Lachnospiraceae*_XPB1014_group, *Staphylococcus*, *Aerococcus*, *Selenomonas*, *Megasphaera*, *Mitsuokella* and *Veillonellaceae*_unclassified.

**Table 1:** **Overview of the main differences in relative abundance of taxa at phylum, family, genus and species level in the** ***Pars oesophagea*** **of the** ***H. suis*** **-positive and -negative pigs**

Level	Taxa	*H. suis* +	*H. suis* −	*p*-value	Corrected *p*-value
Phylum	Fusobacteria	4.20 ± 1.02	1.26 ± 0.51	0.018	0.190
Family	*Fusobacteriaceae*	3.50 ± 0.83	0.94 ± 0.36	0.015	0.060
	*Porphyromonadaceae*	3.25 ± 0.51	0.19 ± 0.08	0.002	0.059
	*Bacteroidaceae*	0.25 ± 0.09	0.03 ± 0.02	0.028	0.206
	*Clostridiaceae*_1	18.02 ± 7.23	3.51 ± 1.44	0.045	0.262
	Gammaproteobacteriaceae*_unclassified*	2.02 ± 0.73	0.35 ± 0.19	0.021	0.177
	*Actinobacteria_unclassified*	0.00 ± 0.00	0.03 ± 0.01	0.005	0.051
	*Bifidobacteriaceae*	0.00 ± 0.00	0.13 ± 0.06	0.005	0.054
	*Corynebacteriaceae*	0.02 ± 0.01	0.27 ± 0.18	0.060	0.285
	*Coriobacteriaceae*	0.01 ± 0.01	0.10 ± 0.05	0.049	0.283
	*Streptococcaceae*	1.62 ± 0.84	2.38 ± 0.98	0.137	0.475
Genus	*Fusobacterium*	3.49 ± 0.83	0.94 ± 0.36	0.009	0.075
	*Porphyromonas*	2.51 ± 1.24	0.17 ± 0.07	0.007	0.073
	*Porphyromonadaceae*_unclassified	0.67 ± 0.34	0.02 ± 0.01	0.042	0.246
	*Bacteroides*	0.25 ± 0.09	0.03 ± 0.02	0.021	0.154
	*Alloprevotella*	3.66 ± 1.58	0.70 ± 0.35	0.034	0.219
	*Clostridium*_sensu_stricto_1	16.93 ± 6.80	3.03 ± 1.19	0.035	0.221
	*Leuconostoc*	0.02 ± 0.01	0.00 ± 0.00	< 0.001	0.019
	*Pasteurella*	0.03 ± 0.01	0.00 ± 0.00	0.003	0.042
	*Gammaproteobacteria*_unclassified	2.02 ± 0.73	0.35 ± 0.19	0.029	0.199
	*Actinobacteria*_unclassified	0.00 ± 0.00	0.03 ± 0.01	0.003	0.040
	*Bifidobacterium*	0.00 ± 0.00	0.13 ± 0.06	0.008	0.072
	Corynebacteriaceae_unclassified	0.00 ± 0.00	0.01 ± 0.00	0.038	0.236
	*Lachnospiraceae*_UCG-007	0.00 ± 0.00	0.02 ± 0.01	< 0.001	0.015
	*Lachnospiraceae*_XPB1014_group	0.00 ± 0.00	0.03 ± 0.02	0.004	0.045
	*Staphylococcus*	0.00 ± 0.00	0.02 ± 0.01	< 0.001	0.054
	*Aerococcus*	0.00 ± 0.00	0.11 ± 0.06	0.009	0.078
	*Selenomonas*	0.00 ± 0.00	0.30 ± 0.15	< 0.001	0.036
	*Megasphaera*	0.02 ± 0.01	1.14 ± 0.70	0.014	0.113
	*Mitsuokella*	0.01 ± 0.01	0.20 ± 0.08	0.011	0.091
	*Veillonellaceae*_unclassified	0.01 ± 0.01	0.42 ± 0.32	0.048	0.272
Species	*Fusobacterium gastrosuis*	3.11 ± 0.78	0.91 ± 0.35	0.015	0.068
	*Porphyromonas*_16S_OTU40	0.96 ± 0.37	0.04 ± 0.02	< 0.001	0.037
	*Porphyromonas*_16S_OTU24	1.36 ± 0.86	0.09 ± 0.04	0.041	0.595
	*Porphyromonas*_16S_OTU293	0.05 ± 0.02	0.01 ± 0.00	0.068	0.817
	*Porphyromonadaceae*_unclassified_16S_OTU1254	0.02 ± 0.01	0.00 ± 0.00	< 0.001	0.349
	*Porphyromonadaceae*_unclassified_16S_OTU61	0.50 ± 0.27	0.01 ± 0.01	0.041	0.600
	*Porphyromonadaceae*_unclassified_16S_OTU212	0.10 ± 0.07	0.00 ± 0.00	0.082	0.930
	*Bacteroides*_16S_OTU103	0.19 ± 0.09	0.02 ± 0.01	0.041	0.598
Species	Bacteroidales_unclassified_16S_OTU977	0.01 ± 0.00	0.00 ± 0.00	0.016	0.335
	*Alloprevotella*_16S_OTU50	0.74 ± 0.37	0.02 ± 0.01	0.026	0.475
	*Alloprevotella*_16S_OTU1438	0.02 ± 0.01	0.00 ± 0.00	0.069	0.822
	*Clostridium*_sensu_stricto_1_GQ249583	7.32 ± 2.86	1.26 ± 0.50	0.045	0.651
	*Leuconostoc*_16S_OTU708	0.02 ± 0.01	0.00 ± 0.00	< 0.001	0.047
	*Pasteurella multocida*	0.03 ± 0.01	0.00 ± 0.00	0.004	0.114
	*Gammaproteobacteria*_unidentified_16S_OTU46	0.30 ± 0.17	1.93 ± 0.69	0.045	0.424
	*Bifidobacterium* AB034094	0.00 ± 0.00	0.07 ± 0.03	0.035	0.537
	*Bifidobacterium thermophilum*	0.00 ± 0.00	0.05 ± 0.03	0.005	0.141
	*Streptococcus*_16S_OTU295	0.01 ± 0.01	0.08 ± 0.04	0.083	0.936
	*Lachnospiraceae*_UCG-007_16S_OTU727	0.00 ± 0.00	0.02 ± 0.01	< 0.001	1.278
	*Lachnospiraceae*_XPB1014_group_AB506358	0.00 ± 0.00	0.03 ± 0.02	0.003	0.093
	*Staphylococcus* EU341210	0.00 ± 0.00	0.01 ± 0.00	0.016	0.304
	*Aerococcus suis*	0.00 ± 0.00	0.09 ± 0.05	0.003	0.092
	*Selenomonas bovis*	0.00 ± 0.00	0.30 ± 0.15	< 0.001	0.046
	*Megasphaera* KF842421	0.00 ± 0.00	0.15 ± 0.08	< 0.001	0.034
	*Megasphaera*_16S_OTU1576	0.00 ± 0.00	0.02 ± 0.01	< 0.001	0.051
	*Megasphaera*_16S_OTU1120	0.00 ± 0.00	0.04 ± 0.01	0.011	0.260
	*Megasphaera elsdenii*	0.01 ± 0.01	0.82 ± 0.53	0.027	0.491
	*Mitsuokella_*16S_OTU1562	0.00 ± 0.00	0.01 ± 0.00	0.031	0.521
	*Mitsuokella* AB506409	0.00 ± 0.00	0.03 ± 0.01	0.041	0.602
	*Mitsuokella* AM500804	0.00 ± 0.00	0.03 ± 0.02	< 0.001	0.060
	*Mitsuokella* DQ797043	0.00 ± 0.00	0.08 ± 0.04	0.033	0.510
	*Veilonellaceae*_unclassified_16S_OTU715	0.00 ± 0.00	0.03 ± 0.01	< 0.001	0.035
	*Veilonellaceae*_unclassfied_16S_OTU1085	0.00 ± 0.00	0.02 ± 0.01	0.049	0.717

The data are presented as the mean relative abundance of the taxa with the standard error of the mean. Statistical differences were calculated using the non-parametric Kruskal–Wallis tests with Tukey post hoc tests and Benjamini–Hochberg False Discovery Rate were performed using STAMP. A corrected *p*-value lower than 0.05 is considered to be significant.

The relative abundance of *F. gastrosuis* was higher in the *Pars oesophagea* of *H. suis*-positive pigs compared to *H. suis*-negative pigs (3% vs 0.9%, *p* = 0.068). This was confirmed by RT-PCR, as *H. suis*-infected pigs showed a significantly higher colonization rate of *F. gastrosuis* in the *Pars oesophagea* compared to non-infected pigs (*p *< 0.05) (Additional file 6D).

### Study 2: *F. gastrosuis* colonization in the porcine gastro-intestinal tract and impact of *H. suis* infection

In the 2–3 months old pigs, the highest abundance of *F. gastrosuis* was found in the oral cavity, followed by the *Pars oesophagea*, independent from the *H. suis* status (*p* < 0.05) (Additional file 7). All pigs’ stomachs tested positive for presence of *F. gastrosuis* and the average number of bacteria per mg tissue was higher in the *Pars oesophagea* than in the other stomach regions (*p *< 0.01) (Additional file 6). The number of *F. gastrosuis* bacteria was lower in the 2–3-compared to the 6–8 months old pigs and adult sows for each stomach region (*p* < 0.01), while no significant differences were observed between the latter groups (Additional file 8).

The number of *F. gastrosuis* bacteria in the oral cavity was highly and positively correlated with the number in the *Pars oesophagea* (*p *< 0.001). The number of *F. gastrosuis* in the *Pars oesophagea* was highly and positively correlated with each of the numbers in the cardiac, fundic and pyloric gland zones, as well as in the jejunum (*p *< 0.001).

The number of *F. gastrosuis* bacteria did not differ between the *H. suis*-negative and -positive 2–3 months old pigs, while a trend was observed towards higher number colonizing the *Pars oesophagea* of *H. suis*-positive 6–8 months old pigs compared to the non-infected age-matched group, although this difference was not significant. The *H. suis*-infected adult sows showed significantly lower number of *F. gastrosuis* colonizing the *Pars oesophagea* compared to the non-infected age-matched group (*p *< 0.05).

#### Gene expression analysis of markers for inflammation and ulceration

Despite several attempts, including the use of different primer pairs, no mRNA expression was detected of the genes encoding claudin 4, NOX2, IL-6 and TNFα in the porcine *Pars oesophagea*.

Compared to the *H. suis*-negative pigs, the mRNA expression of claudin 18, Hsp 72 and IL-8 was upregulated in the *Pars oesophagea* of *H. suis*-positive pigs, although either borderline or not significantly (*p* = 0.050, 0.211 and 0.139, respectively). In contrast, claudin 2, claudin 3 and CXCL2 gene expressions were either significantly or not significantly downregulated (*p* = 0.043, 0.093 and 0.065, respectively) (Additional files 9, 10).

The fold changes of claudin 18, Hsp 72, keratin 6A and ZO1 were more pronounced in pigs with a high number of *F. gastrosuis* bacteria per mg gastric tissue of the *Pars oesophagea*, since positive correlations were found between both. Conversely, the fold changes of claudin 3, BFGF, CXCL2 and IL-1β were less pronounced in pigs with a high number of *F. gastrosuis* bacteria per mg gastric tissue due to negative correlations between both (Additional file 11).

### Study 3: Cell-death inducing effects of *F. gastrosuis* on epithelial neoplastic cell lines

*Fusobacterium gastrosuis* was able to survive in an aerobic environment up to 12 h. After this incubation time, the viability of *F. gastrosuis* was significantly decreased and after 36 h incubation, viable *F. gastrosuis* bacteria could no longer be detected (data not shown).

#### Cell-death inducing effects on *F. gastrosuis* on MKN-7 cell line

**Figure 2:**
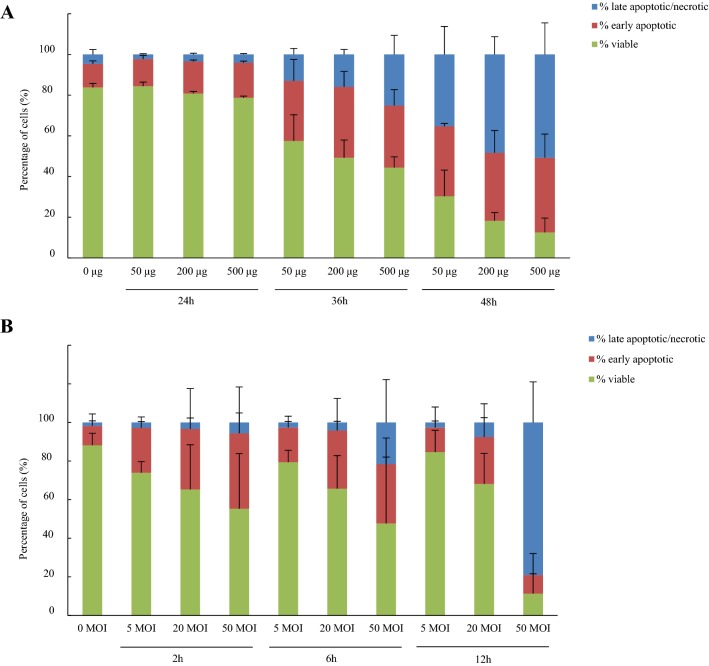
**Percentage of viable, early apoptotic, late apoptotic/necrotic MKN-7 cells after incubation with different concentrations of** ***F. gastrosuis*** **lysate (A) and viable** ***F. gastrosuis*** **bacteria (B).** Data are shown as the average (4 strains, 3 replications per strain) percentages of viable, early apoptotic and late apoptotic/necrotic MKN-7 cells with standard deviation. **A** The cells were incubated for 24, 36 and 48 with 50, 200 and 500 µg *F. gastrosuis* lysate (4 strains). **B** The cells were incubated for 2, 6 and 12 h with 5, 20 and 50 MOI viable *F. gastrosuis* bacteria (4 strains).

Compared to the negative control, the viability of MKN-7 cells hardly decreased with an average of 3% after 24 h incubation with the different concentrations of *F. gastrosuis* lysate, but significantly decreased with an average of 32% and 65% after 36 h and 48 h incubation, respectively (*p *< 0.05) (Figure 2A). After 2 h, 6 h and 12 h incubation with the different concentrations of viable *F. gastrosuis* bacteria, cell viability significantly decreased with an average of 23%, 24% and 34%, respectively, compared to the negative control (*p *< 0.05) (Figure 2B).

Overall, the cell viability significantly decreased with increasing concentrations of *F. gastrosuis* lysate and MOI as well as with prolonged incubation time compared to the negative control (*p *< 0.05) (Additional files 12, 13 and 14). Although the observed decreases in cell viability were systematically caused by both an increase in apoptosis and late apoptosis/necrosis, the percentage of early apoptotic cells increased after short incubation time and/or low concentrations of *F. gastrosuis* lysate and MOI, while the percentage of late apoptotic/necrotic cells increased after longer incubation time and/or higher concentrations of *F. gastrosuis* lysate and MOI (Additional files 12, 13 and 14). Morphological features of apoptosis (i.e. plasma membrane blebbing, pyknosis) and necrosis (i.e. cell swelling, cytoplasmic vacuoles) reflecting these differences were observed on Hemacolor staining (Additional file 14).

Compared to *F. gastrosuis* lysate, more variation was observed between the four *F. gastrosuis* strains after incubation with viable *F. gastrosuis* bacteria. CDW1 lysate had the strongest effect on cell viability, CDW3 the weakest (*p *< 0.05). Conversely, viable CDW1 bacteria had the weakest effect on cell viability, CDW6 the strongest (*p *< 0.05).

In general, cell viability was significantly higher after incubation with the four *F. gastrosuis* strains compared to the positive control *F. necrophorum* subsp. *necrophorum* (*p *< 0.05). However, a significantly lower viability was observed for following conditions: at 50 µg lysate after 24 h, 36 h and 48 h for CDW1 and CDW3, and after 48 h also for CDW6 and CDW8; at 200 µg lysate CDW1, CDW6 and CDW8 after 48 h; at 50 MOI viable bacteria after 6 h and 12 h for CDW1 and after 12 h also for CDW6 and CDW8 (*p *< 0.05).

#### Cell-death inducing effects on *F. gastrosuis* on KYSE-450 cell line

**Figure 3:**
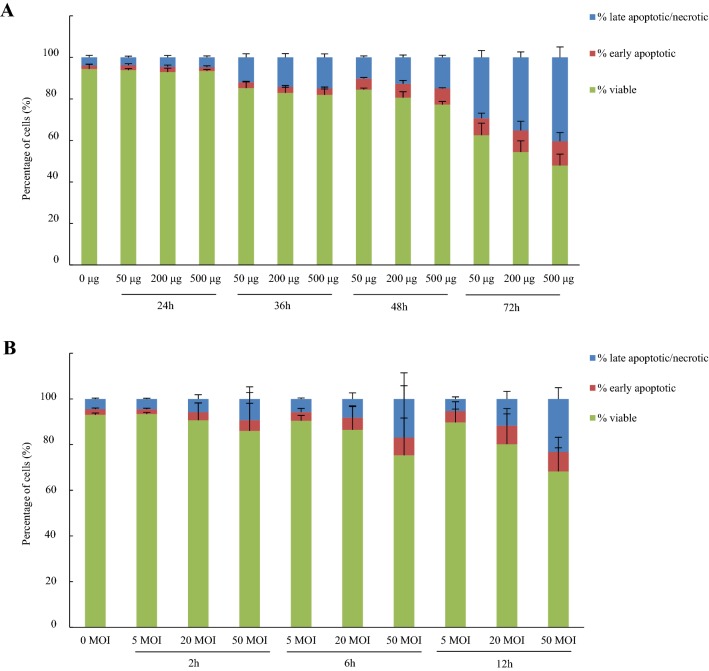
**Percentage of viable, early apoptotic, late apoptotic/necrotic KYSE-450 cells after incubation with different concentrations of** ***F. gastrosuis*** **lysate (A) and viable** ***F. gastrosuis*** **bacteria (B).** Data are shown as the average (4 strains, 3 replications per strain) percentages of viable, early apoptotic and late apoptotic/necrotic KYSE-450 cells with standard deviation. **A** The cells were incubated for 24, 36, 48 and 72 h with 50, 200 and 500 µg *F. gastrosuis* lysate (4 strains). **B** The cells were incubated for 2, 6 and 12 h with 5, 20 and 50 MOI viable *F. gastrosuis* bacteria (4 strains). The percentage late apoptotic/necrotic cells (i.e. Annexin-V-FITC positive and PI positive) contains a small subpopulation of Annexin-V-FITC negative and PI positive cells defined as late necrotic cells. See Additional files 15, 16 for more detailed information.

The viability of KYSE-450 cells was significantly decreased with an average of 4%, 10%, 14% and 37% after 24 h, 36 h, 48 h and 72 h incubation, respectively, with the different concentrations of *F. gastrosuis* lysate compared to the negative control (*p *< 0.05) (Figure 3A). Compared to the negative control, cell viability hardly decreased with an average of 3% after 2 h incubation with the different concentrations of viable *F. gastrosuis* bacteria, but significantly decreased with an average of 8% and 15% after 6 h and 12 h incubation, respectively (*p *< 0.05) (Figure 3B).

Overall, the cell viability significantly decreased with increasing concentrations of *F. gastrosuis* lysate and MOI as well as with prolonged incubation time compared to the negative control (*p *< 0.05) (Additional files 15, 16 and 17). Although the observed decreases in cell viability were systematically caused by both an increase in early apoptosis and late apoptosis/necrosis, the percentage of early apoptotic cells increased after short incubation time and/or low concentrations of *F. gastrosuis* lysate and MOI, while the percentage of late apoptotic/necrotic cells increased after longer incubation time and/or higher concentrations of *F. gastrosuis* lysate and MOI. Morphological features of apoptosis (i.e. plasma membrane blebbing) and necrosis (i.e. cell swelling, cytoplasmic vacuoles) reflecting these differences were observed on Hemacolor staining (Additional file 17).

In contrast to the MKN-7 cell line, a small subpopulation of Annexin-V-FITC negative and PI positive KYSE-450 cells was detected after incubation with *F. gastrosuis* viable bacteria and lysates. This subpopulation was defined as late necrotic and increased with increasing concentrations of *F. gastrosuis* lysate and MOI as well as with prolonged incubation time compared to the negative control (*p *< 0.05) (Additional files 15, 16 and 17).

A similar effect on cell viability was seen after incubation with lysate obtained from the different *F. gastrosuis* strains. Conversely, more variance was seen after incubation with viable *F. gastrosuis* bacteria, where CDW1 had the weakest effect on cell viability and CDW6 the strongest (*p *< 0.05).

In general and in contrast to the MKN-7 cell line, the cell viability was lower after incubation with the four *F. gastrosuis* lysates compared to the positive control *F. necrophorum* subsp. *necrophorum*. A significantly lower viability was observed for the following conditions: at 50 µg lysate after 24 h and 72 h for CDW1, CDW3, CDW6 and CDW8, after 36 h and 48 h also for CDW6 and after 48 h also for CDW1 and CDW3; at 200 µg lysate after 24 h, 48 h and 72 h for CDW1 and CDW3 and after 72 h also CDW6 and CDW8; at 500 µg lysate after 24 h, 48 h and 72 h for CDW1 and after 48 h and 72 h also for CDW3 and CDW6 (*p *< 0.05).

In general and similar to the MKN-7 cell line, the cell viability was significantly higher after incubation with the four viable *F. gastrosuis* strains compared to the positive control *F. necrophorum* subsp. *necrophorum* bacteria (*p* < 0.05). However, a significantly lower viability was observed for following conditions: at five MOI viable bacteria after 6 h for CDW3 and CDW6 and after 12 h also for CDW6; at 20 MOI viable bacteria after 6 h and 12 h for CDW6; at 50 MOI after 6 h for CDW6 and after 12 h also for CDW3 (*p *< 0.05).

#### Properties of cell death inducing *F. gastrosuis* component(s)

Heat treatment completely abolished the cell-death inducing capacity of the four *F. gastrosuis* viable bacteria and lysates. Proteinase K treatment completely abolished the cell-death inducing capacity of viable *F. gastrosuis* bacteria, while no effects were seen for the trypsine and pronase treatments. Variable effects were seen for the paraformaldehyde treatment, namely a complete loss, partial loss (10–30%) or no loss of cell-death inducing capacities, and this independent from the strains (Table 2). *F. gastrosuis* bacteria remained viable after the different protease treatments and bacterial growth was similar to the untreated *F. gastrosuis* bacteria, while paraformaldehyde and heat treatment resulted in complete loss of viability.

**Table 2:** **Overview of the effect of heat, protease and formaldehyde treatment on cell-death inducing capacity of the 4** ***F. gastrosuis*** **strains**

Treatment	Loss of cell-death inducing capacity	
	*F. gastrosuis* viable bacteria	*F. gastrosuis* lysate
Heat	+	+
Proteinase K	+	/
Trypsine	−	/
Pronase	−	/
Paraformaldehyde	±	/

+: complete loss of cell-death inducing capacity, −: no loss of cell-death inducing capacity, ±: variable effects on cell-death inducing capacity, /: not determined.

#### Activation of the pyroptotic pathway

All samples tested negative for presence of pro- and active IL-1β and caspase-1 proteins on western blot analysis, while bands were obtained for the positive controls (data not shown).

### Study 4: Potential virulence associated genes in *F. gastrosuis*

A search for DEG homologous sequences in the proteome of *F. gastrosuis* yielded 1048 hits. Obtained hits have been reported to be involved in structural organization, nutrient uptake, pathogenesis, antibiotic resistance and other processes essential for the survival of *F. gastrosuis* (Additional file 18).

Using the VirulentPred tool, 922 genes, of which 370 encoded hypothetical proteins, were predicted to be associated with virulence. Of the entire *F. gastrosuis* proteome, 51 proteins showed a close match with Pfam domains associated with virulence, antibiotic resistance and immune-evasion (Additional file 18). About 162 proteins of the proteome showed significant hits against VFDB. The obtained hits have been reported to be involved in adhesion, invasion, immune-evasion, capsule formation, resistance to antibiotics and uptake of magnesium, zinc and iron (Additional file 18). Some examples of these potential virulence factors are: (i) hemolysin—lyses erythrocytes and creates anaerobic environment at the site of infection, stimulates the production of IL-1β and TNFα; (ii) ABC transporters—facilitates iron uptake, related with antibiotic resistance; (iii) lipooligosaccharides synthesis—involved in immune system evasion, attachment to epithelial tissue, mediator of the proinflammatory response; (iv) cytolysin—forms pores in cell membrane, induces apoptosis, promotes cellular invasion, triggers iNOS and cytokine release (Additional file 18). In total, 232 proteins showed a close match with virulence factors of other *Fusobacterium* spp. reported in the literature. Some examples of these matches are: (i) immunosuppressive protein (fipA)—inhibits T-cell activation; (ii) haemagglutinin—attachment to epithelial cells; (iii) outer membrane proteins—mediate adherence to other pathogenic bacteria and host cells, suppress host immune system, induce cell death in lymphocytes; (iv) serine protease—degrades the extracellular matrix proteins fibrinogen and fibronectin as well as collagen I and collagen IV, contribute to damage of periodontal tissues, helps the evasion of the immune system. Finally, *F. gastrosuis* showed presence of genes associated with active invasion of host cells (Additional file 18).

## Discussion

The gastro-intestinal microbiota plays an important role in host metabolism, nutrition, immunity and protection against pathogens [26, 27]. Disturbance of this delicate system is strongly correlated with the onset and progression of a wide range of pathologies [28]. Alterations in the gastric microbial community may play a role in porcine gastric lesion development [1]. However, due to technical limitations and the fact that the stomach was long considered inhospitable, limited information is available on the gastric microbiota composition of pigs [29]. For the first time, the microbiota of the upper, non-glandular part of the stomach of 6–8 months old pigs was determined, as well as the impact on it of a natural *H. suis* infection.

Proteobacteria, Firmicutes, Bacteroidetes, Fusobacteria and Actinobacteria were the most abundant phyla present in the *Pars oesophagea* of 6–8 months old pigs, irrespective of the *H. suis* status. These phyla have also been shown to dominate the gastric microbiota of weaned pigs [30, 31]. Nevertheless, their relative abundance may differ, as Proteobacteria was the most dominant phylum in the *Pars oesophagea* of 6–8 months old pigs in the current study, while Firmicutes has been shown to dominate this stomach region in weaned pigs [31]. This apparent discrepancy between both studies might be explained by the age difference of the pigs. In suckling pigs, *Lactobacillus* spp., which form a major part of the Firmicutes phylum, colonize the upper, non-glandular part of the stomach in large numbers. After weaning, the diet changes from sow milk to hard food, resulting in a gradual decrease of *Lactobacillus* spp. [30]. Indeed, diet can greatly affect the microbial community, as bacterial enterotypes are known to cluster based on the dietary abundance of animal protein relative to carbohydrate [28]. Proteobacteria have also been shown to dominate stomach content as well as the cardiac, fundic and pyloric mucosa of weaned pigs, suggesting that other factors may affect the relative abundance of phyla, such as management and sampling sites or techniques [29]. Furthermore, the microbiota composition can also vary substantially within different herds and within pigs from the same herd, especially at the lower taxonomic levels. These factors complicate both the straightforward comparison of study results and the unequivocal definition of the gastric microbial community of healthy pigs [28, 32].

*Fusobacteriaceae* are part of the core microbiota of the soft palate tonsils in pigs [33, 34] and some members, such as *F. necrophorum,* have been associated with stomatitis and tooth root abscesses [35]. In the current study on the group of the 2–3 months old pigs, the highest colonization rate of *F. gastrosuis* was found in the oral cavity, indicating that the oropharynx, and potentially its tonsils, may function as a natural reservoir for this bacterium. *F. gastrosuis* may enter the stomach through ingestion of food, water or saliva, after which it may opportunistically colonize the upper, non-glandular *Pars oesophagea*. Future studies are necessary to verify if *F. gastrosuis* does belong to the oral microbiota and/or if it may induce oropharyngeal pathologies. Interestingly, recent metagenomic studies have also shown presence of *F. gastrosuis* in the nasal and oral microbiota of dogs (Bernard Taminiau, 2018, personal communication) and in the faeces of humans [36], indicating that *F. gastrosuis* is able to colonize a wide range of mammalian hosts, as has been described for *F. nucleatum* and *F. necrophorum* [6]. Nevertheless, although unlikely, it might still be possible that *F. gastrosuis* was merely transient in these three hosts.

Recent studies indicate that *H. suis* infection plays an important role in porcine gastric ulceration, probably by affecting gastric acid secretion and influencing the gastric microbiota [4, 37]. In these studies, also high numbers of *F. gastrosuis* were detected in the gastric microbial community of *H. suis*-infected 6–8 months old pigs with a downregulation of markers for gastric acid secretion [4, 5]. In the present study, *16S rRNA* gene amplicon pyrosequencing revealed a higher relative abundance of *F. gastrosuis* in the *Pars oesophageal* microbiota of *H. suis*-infected 6–8 months old pigs compared to non-infected pigs which was confirmed by RT-PCR. Analysis of many more pig stomachs of the same age group by RT-PCR showed a high *F. gastrosuis* colonization rate in the *Pars oesophagea* of *H. suis*-infected pigs as well, although the difference with non-infected pigs became less pronounced. This apparent discrepancy may have been caused by variation between herds or other factors such as diet, feeding strategy and management. Nonetheless, more stomachs should be investigated to confirm the increased *F. gastrosuis* colonization rate in *H. suis*-infected pigs. In marked contrast with our group of 6–8 months old pigs, the numbers of *F. gastrosuis* bacteria did not differ between *H. suis*-infected and non-infected 2–3 months old pigs. This may have been caused by the absence of gastric acid secretion alterations during the first phase of a *H. suis* infection i.e. in the 2–3 months old pigs as previously reported [4]. The lower numbers of *F. gastrosuis* colonizing the *Pars oesophagea* of *H. suis*-infected adult sows compared to non-infected sows might be a consequence of the upregulation of gastric acid secretion during the chronic phase of infection i.e. in the *H. suis*-infected adult sows as again previously reported [4]. Combining the results of the current and our previous studies, we hypothesize that during the earlier phases of a *H. suis* infection (i.e. in the 6–8 months old pigs), gastric acid secretion is downregulated, resulting in higher numbers of *F. gastrosuis* in the non-glandular part of the stomach which may facilitate the initiation of lesion development. During the more chronic phase of the infection (i.e. in the adult sows), gastric acid secretion is stimulated which may further aggravate lesion severity in the non-glandular part of the stomach which is not protected against acid due to the lack of mucus. Combined *F. gastrosuis* and *H. suis* infections in an experimental model complementing the observations obtained in naturally infected animals are, however, necessary to confirm or reject this working hypothesis.

Since few commercial porcine gastric cell line exists and as the epithelium of the oesophagus from humans and the *Pars oesophagea* of pigs is very similar [38], human-derived cell lines were used in the present study to further investigate in vitro the pathogenic traits of *F. gastrosuis*. Overall, cell death was clearly induced by both the viable *F. gastrosuis* bacteria and their lysate in the two selected cell lines. As lesions of the *Pars oesophagea* are characterized by the presence of swollen epithelial cells and necrotic debris [39, 40], these in vitro cell death data further highlight a potential role of *F. gastrosuis* in the development of porcine gastric ulceration. Finally, it would be interesting to evaluate the effect of *F. gastrosuis* on mucosal explants of the porcine *Pars oesophagea*, as these target species explants comprise several additional host cell and host cell-environment interactions lacking in the relatively simple gastric cell line set-up that may be important in the elucidation of porcine gastric ulceration development.

In contrast to the cell death mechanism reported for *F. nucleatum* in a human gingival cell line [21], the current study did not observe indications of pyroptotic pathway (i.e. pro-inflammatory programmed cell death) activation in epithelial cells incubated with *F. gastrosuis*, characterized by the absence of the pro-inflammatory caspase-1 and IL-1β specific protein bands. It is possible that other caspases were activated through either the intrinsic or extrinsic apoptotic pathway. These complex cell-death inducing pathways needs to be further elucidated, for example by analyzing the expression of different caspase family members through flow cytometry [41].

The mechanisms by which *F. gastrosuis* induces host cell death are unknown. Our data suggest that heat labile proteins most likely play a role, as heat- and proteinase K-treated *F. gastrosuis* completely lost their cell death inducing potential. In contrast, pronase and trypsin treatments did not affect this cell death inducing capacity of *F. gastrosuis,* similar as described for other *Fusobacterium* spp. [42]. This may be explained by differences in enzymatic specificity, as trypsin specifically cleaves the peptide bonds at the carboxyl terminus of lysine and arginine residues, while proteinase K non-specifically cleaves at aromatic, aliphatic as well as hydrophobic amino acid residues [43]. The activity of pronase is also broad, yet the cell-death inducing capacity of *F*. *gastrosuis* was unaffected after the latter treatment. We hypothesize that other, currently unknown, factors may have further contributed here. For example, genes associated with capsule and biofilm formation as found in the proteome of *F. gastrosuis* may have contributed to this resistance against proteases such as pronase. As for the effect of formaldehyde treatment, widely used as fixation agent which slowly cross-links proteins in the bacterial cell wall, variable results were obtained. This indicates that cell-death inducing metabolites are, at least partly, associated with the bacterial surface and that their expression may be affected by environmental signals such as cell-contact. Nevertheless, this interpretation should be made with caution.

The current study identified that outer membrane proteins (OMPs) homologous to autotransporter secretion systems (type V) and/or ABC transporters are present in the proteome of *F. gastrosuis.* In analogy with a previous study, these OMPs might induce cell-death [44]. Indeed, bacterial secretion systems are associated with host cell death through active transport of effector metabolites [45] like hemolysin, leukotoxin and protease, all pore-forming proteins that either directly destroy host cells at high concentrations or activate apoptosis through induction of host plasma membrane changes at lower concentrations. The presence of serine protease and hemolysin homologs in the proteome of *F. gastrosuis,* as well as an increased percentage necrosis when epithelial cells were incubated with high *F. gastrosuis* concentrations support their potential role in the induction of cell death. Other putative virulence factors present in the proteome of *F. gastrosuis* may also have contributed to host cell death. Homologs of methionine gamma lyase, cystathionine gamma- and beta lyase were found to be associated with host tissue destruction through production of volatile sulfur products [46, 47], while gamma-glutamyl transferase [19] and genes associated with butyrate production [25] have both been associated with host cell death through the production of reactive oxygen species (ROS). Last but not least, some *Fusobacterium* spp. possess genes encoding a diverse set of adhesins and membrane-related proteins allowing to actively enter host cells even in the absence of tissue damage [6]. Analysis of the *F. gastrosuis* genome revealed the presence of such genes, supporting host colonization, host immune system evasion and deeper tissue penetration. Additional studies are necessary to determine if these putative virulence factors are expressed, secreted as well as functional. It would be interesting to create mutants lacking genes encoding these putative virulence-associated factors in order to investigate their roles in the pathogenic significance of *F. gastrosuis*.

In conclusion, high numbers of *F. gastrosuis* were demonstrated in the oropharynx and *Pars oesophagea* of pigs. It was also demonstrated that *F. gastrosuis* induces epithelial cell death and that genes are present in the genome of this bacterium with sequence similarity to genes encoding factors involved in adhesion, invasion and induction of cell death as well as immune evasion. We hypothesize that, in a gastric environment altered by *H. suis*, colonization and invasion of the *Pars oesophagea* and production of epithelial cell death inducing metabolites by *F. gastrosuis* cause ulceration. Experimental studies in pigs infected with *H. suis* and *F. gastrosuis* are necessary to confirm this hypothesis.

## Additional files

**Additional file 1.** **List of primers used in quantitative RT-PCR for gene expression analysis of porcine housekeeping genes and markers for inflammation, hyperkeratosis and ulceration.**

**Additional file 2.** **Gating strategy of the MKN-7 cell line.** (A) FSC-A/SSC-A represents the distribution of cells in the light scatter based on their size and intracellular complexity, respectively. The cells of interest are gated excluding debris. (B) FSC-A/FSC-H allows discrimination between single cells and doublets, single cells are gated. (C) Gain settings and compensation matrix. (D-E) FITC-A/PE-A identifies the selective subpopulations: viable (Annexin-V-FITC negative, PI negative), early apoptotic (Annexin-V-FITC positive, PI negative) and late apoptotic/necrotic (Annexin-V-FITC positive, PI positive) cells.

**Additional file 3.** **Gating strategy of the KYSE-450 cell line.** (A) FSC-A/SSC-A represents the distribution of cells in the light scatter based on their size and intracellular complexity, respectively. The cells of interest are gated excluding debris. (B) FSC-A/FSC-H allows discrimination between single cells and doublets, single cells are gated. (C) Gain settings and compensation matrix. (D-E) FITC-A/PE-A identifies the selective subpopulations: viable (Annexin-V-FITC negative, PI negative), early apoptotic (Annexin-V-FITC positive, PI negative), late apoptotic/necrotic (Annexin-V-FITC positive, PI positive) and late necrotic (Annexin-V-FITC negative, PI positive) cells.

**Additional file 4.** **Bacterial community compositions present in the** ***Pars oesophagea*** **of each individual pig.** The cumulated histograms show the relative abundance of the identified taxa at phylum (A), family (B) and genus (C) level. At family and genus level, taxa with a relative abundance <1% are merged in the category “others”. 1–10 = *H. suis-*negative pigs, 11–20 = *H. suis-*positive pigs. The unclassified populations correspond to defined groups of the genus level for which a taxonomical classification assignation to the genus cannot be attributed. These populations are therefore labelled with the first defined superior hierarchical taxonomic level followed by “_unclassified” to prevent confusion.

**Additional file 5.** **Overview of the bacterial richness (A), diversity (B) and evenness (C) of the** ***Pars oesophagea*** **of** ***H. suis-*positive and -negative pigs.** The data are represented as scatter plots: each dot represents a pig, the middle line represents the median and the whiskers represent the standard error or the mean.

**Additional file 6.** **The number of** ***F. gastrosuis*** **bacteria in the different stomach regions of** ***H. suis-*positive and -negative 2–3 months old pigs (A), 6–8 months old pigs (B), adult sows (C) and the pigs used for the metagenomics study (D).** Data are shown as log10 values of the average number of *F. gastrosuis* bacteria per mg tissue with standard deviation. Statistical differences were calculated using the non-parametric Kruskal-Wallis H test. *, *p* < 0.05.

**Additional file 7.** **The number of** ***F. gastrosuis*** **bacteria in the oral cavity and gastro-intestinal tract of 2–3 months old pigs.** Data are shown as log10 values of the average number of *F. gastrosuis* bacteria per mg tissue with standard deviation. Statistical differences were calculated using the non-parametric Kruskal-Wallis H test. *, *p* < 0.05; **, *p* < 0.001 significant differences between the regions.

**Additional file 8.** **The number of** ***F. gastrosuis*** **bacteria in the different stomach regions of 2–3 months old pigs, 6–8 months old pigs and adult sows.** Data are shown as log10 values of the average number of *F. gastrosuis* bacteria per mg tissue with standard deviation. Statistical differences were calculated using the non-parametric Kruskal-Wallis H test. *, *p* < 0.05; **, *p* < 0.001 significant differences between the stomach regions. Significant differences between the age groups are indicated with brackets.

**Additional file 9.** **General overview of gene expression analysis of markers for inflammation and ulceration in the** ***Pars oesophagea*** **of** ***H. suis*-negative and -positive pigs.** The data are presented as fold changes in gene expression normalized to 3 reference genes and relative to a *H. suis*-negative control pigs. The fold changes are shown as means with the standard error of the mean. Statistical differences were calculated using the non-parametric Kruskal-Wallis H test. *, *p* < 0.05; significant differences between the *H. suis-*positive pigs and -negative pigs.

**Additional file 10.** **Overview of relative fold changes of altered markers for inflammation and ulceration in the** ***Pars oesophagea*** **of** ***H. suis*-positive pigs.** The data are presented as fold changes in gene expression normalized to 3 reference genes and relative to the *H. suis-*negative pigs. The fold changes are shown as means with the standard error of the mean. Statistical differences were calculated using the non-parametric Kruskal-Wallis H test. A *p*-value lower than 0.05 is considered to be significant.

**Additional file 11.** **Correlation between the number of colonizing** ***F. gastrosuis*** **bacteria per mg gastric tissue and the expression of markers for inflammation and ulceration.** The data are presented as a scatter plot: each dot represents the individual data of a pig. The trendline shows the relationship between the relative mRNA expression of a marker for inflammation/ulceration and the log10 values of the number of *F. gastrosuis* bacteria per mg gastric tissue. y = equation of the trendline. *r* = Pearson correlation coefficient, calculated using SPSS Statistics 24. A *r*-value close to 1 indicates a strong, positive correlation, whereas a *r*-value of −1 indicates a strong, negative correlation. *P*-values lower than 0.05 are considered to be significant.

**Additional file 12.** **Percentage of viable, early apoptotic and late apoptotic/necrotic MKN-7 cells after incubation with** ***F. gastrosuis*** **lysate.** Data are shown as the average (*n* = 3) percentages of viable (green), early apoptotic (red) and late apoptotic/necrotic (blue) MKN-7 cells with standard deviation. The cells were incubated for 24 (A), 36 (B) and 48h (C) with 50 µg, 200 µg and 500 µg *F. gastrosuis* lysate (4 strains, CDW1, 3, 6 and 8) and *F. necrophorum* subsp. *necrophorum* (Fnn) as positive control. * Significant differences between the negative control and cells incubated with each bacterial lysate (*p* < 0.05).

**Additional file 13.** **Percentage of viable, early apoptotic and late apoptotic/necrotic MKN-7 cells after incubation with viable** ***F. gastrosuis*** **bacteria.** Data are shown as the average (*n* = 3) percentages of viable (green), early apoptotic (red) and late apoptotic/necrotic (blue) MKN-7 cells with standard deviation. The cells were incubated for 2 (A), 6 (B) and 12h (C) with 5 MOI, 20 MOI and 50 MOI viable *F. gastrosuis* bacteria (4 strains, CDW1, 6, 6 and 8) and *F. necrophorum* subsp. *necrophorum* (Fnn) as positive control. * Significant differences between the negative control and cells incubated with each bacterial strain (*p* < 0.05).

**Additional file 14.** **Visualization of MKN-7 cellular morphology using hemacolor staining and detection of early apoptotic and late apoptotic/necrotic cells using flow cytometry.** (A-D) Hemacolor staining of MKN-7 cells incubated (A) without *F. gastrosuis* for 48h and (B-D) with 500 µg *F. gastrosuis* strain CDW1 incubated for (B) 24 h, (C) 36 h and (D) 48h. Following morphologic features can be seen: plasma membrane blebbing (white arrow), cell swelling (white star), pyknosis (black arrow), cytoplasmic vacuoles (black star). Original magnification x400, scale bar represents 10 µm. (E-L) Representative population plots displaying viable (green, Annexin-V-FITX negative, PI negative), early apoptotic (red, Annexin-V-FITX positive, PI negative), late apoptotic/necrotic (blue, Annexin-V-FITX positive, PI positive) cells of MKN-7 cells incubated (E) without *F. gastrosuis* lysate for 48 h; (F-H) with 500 µg *F. gastrosuis* strain CDW1 for (F) 24 h; (G) 36 h and (H) 48 h; (I) without viable *F. gastrosuis* bacteria for 12h; (J-L) with 50 MOI *F. gastrosuis* bacteria strain CDW8 for (J) 2h; (K) 6h and (L) 12h. Y-axis: propidium iodide (PE) signal intensity; X-axis: Annexin-V-fluorescein isothiocyanate (FITC) signal intensity. The percentage of population plots is presented in the corresponding gate.

**Additional file 15.** **Percentage of viable, early apoptotic, late apoptotic/necrotic and late necrotic KYSE-450 cells after incubation with** ***F. gastrosuis*** **lysate.** Data are shown as the average (*n* = 3) percentages of viable (green), early apoptotic (red), late apoptotic/necrotic (blue) and late necrotic (orange) KYSE-450 cells with standard deviation. The cells were incubated for 24 (A), 36 (B), 48 (C) and 72 h (D) with 50 µg, 200 µg and 500 µg *F. gastrosuis* lysate (4 strains, CDW1, 3,6 and 8) and *F. necrophorum* subsp. *necrophorum* (Fnn) as positive control. * Significant differences between the negative control and cells incubated with each bacterial lysate (*p* < 0.05).

**Additional file 16.** **Percentage of viable, early apoptotic, late apoptotic/necrotic and late necrotic KYSE-450 cells after incubation with viable** ***F. gastrosuis*** **bacteria.** Data are shown as the average (*n* = 3) percentages of viable (green), early apoptotic (red), late apoptotic/necrotic (blue) and late necrotic (orange) KYSE-450 cells with standard deviation. The cells were incubated for 2 (A), 6 (B) and 12 h (C) with 5 MOI, 20 MOI and 50 MOI viable *F. gastrosuis* bacteria (4 strains, CDW1, 3, 6 and 8) and *F. necrophorum* subsp. *necrophorum* (Fnn) as positive control. * Significant differences between the negative control and cells incubated with each bacterial strain (*p* < 0.05).

**Additional file 17.** **Visualization of KYSE-450 cellular morphology using hemacolor staining and detection of early apoptotic and late apoptotic/necrotic cells using flow cytometry.** (A-D) Hemacolor staining of KYSE-450 cells incubated (A) without *F. gastrosuis* for 48 h and (B-D) with 500 µg *F. gastrosuis* strain CDW1 incubated for (B) 36 h, (C) 48 h and (D) 72 h. Following morphologic features can be seen: plasma membrane blebbing (white arrow), cell swelling (white star), cytoplasmic vacuoles (black star). Original magnification x400, scale bar represents 10 µm. (E-M) Representative population plots displaying viable (green, Annexin-V-FITC negative, PI negative), early apoptotic (red, Annexin-V-FITC positive, PI negative), late apoptotic/necrotic (blue, Annexin-V-FITC positive, PI positive) cells and late necrotic (orange, Annexin-V-FITC negative, PI positive) of KYSE-450 cells incubated (E) without *F. gastrosuis* lysate for 72 h; (F-I) with 500 µg *F. gastrosuis* strain CDW1 for (F) 24 h; (G) 36 h; (H) 48 h and (I) 72 h; (J) without viable *F. gastrosuis* bacteria for 12 h; (K-M) with 50 MOI *F. gastrosuis* bacteria strain CDW8 for (K) 2 h; (L) 6h and (M) 12 h. Y-axis: propidium iodide (PE) signal intensity; X-axis: Annexin-V-fluorescein isothiocyanate (FITC) signal intensity. The percentage of population plots is presented in the corresponding gate.

**Additional file 18.** **Overview of potential virulence associated genes in** ***F. gastrosuis*** **using DEG, VirulentPred tool, Pfam and literature.**
