# Supplementary material for: Characterization of the non-glandular gastric region microbiota in Helicobacter suis-infected versus non-infected pigs identifies a potential role for Fusobacterium gastrosuis in gastric ulceration
Source: Vet Res. 2019 May 24;50:39. doi: 10.1186/s13567-019-0656-9 (PMC6534906; doi:10.1186/s13567-019-0656-9)
Supplement: Supplementary file 1 — Additional file 1. List of primers used in quantitative RT-PCR for gene expression analysis of porcine housekeeping genes and markers for inflammation, hyperkeratosis and ulceration. [file 13567_2019_656_MOESM1_ESM.docx]

**Additional file 1**: List of primers used in quantitative RT-PCR for gene expression analysis of porcine housekeeping genes and markers for inflammation, hyperkeratosis and ulceration.

| **Primer** | **Sequence** | **Reference** |
| --- | --- | --- |
| HPRT-forward | 5’-GGA CTT GAA TCA TGT TTG TG-3’ | [18] |
| HPRT-reverse | 5’-CAG ATG TTT CCA AAC TCA AC-3’ | [18] |
| Cyc-forward | 5’-CCT GAA CAT ACG GGT CCT G-3’ | [3] |
| Cyc-reverse | 5’-AAC TGG GAA CCG TTT GTG TTG-3’ | [3] |
| HMBS-forward | 5’-AGG ATG GGC AAC TCT ACC TG-3’ | [18] |
| HMBS-reverse | 5’-GAT GGT GGC CTG CAT AGT CT-3’ | [18] |
| RPL4-forward | 5’-CAA GAG TAA CTA CAA CCT TC-3’ | [18] |
| RPL4-reverse | 5’-GAA CTC TAC GAT GAA TCT TC-3’ | [18] |
| Claudin 1-forward | 5’-GAT GCG GAT GGC TGT CAT TG-3’ | This study |
| Claudin 1-reverse | 5’-CCA GAA GGC AGA GAG AAG CA-3’ | This study |
| Claudin 2-forward | 5’-GGC TCT CTA CTT GGG CAT CA-3’ | This study |
| Claudin 2-reverse | 5’-ACT CTT GGC TTT GGG TGG TT-3’ | This study |
| Claudin 3-forward | 5’-GCC AAA GCC AAG ATC CTC TAC-3’ | This study |
| Claudin 3-reverse | 5’-GGA CTG GTC TCG GAT GCA A-3’ | This study |
| Claudin 4-forward | 5’-GAC TCA CCG GAA GCT GTG TTC-3’ | This study |
| Claudin 4-reverse | 5’-GAA GAG AGG CTT TTC ACC GC-3’ | This study |
| Claudin 18-forward | 5’-CCC TGA TGA TCG TGG GCA TA-3’ | This study |
| Claudin 18-reverse | 5’-ACT CCA GCG ATT GTG CAA AG-3’ | This study |
| Keratin 6-forward | 5’-CTC AGG AGT AAC CCA GTA CCA-3’ | This study |
| Keratin 6-reverse | 5’-TTG GTG TCC AGG ACC TTG TT-3’ | This study |
| Heat shock protein 27-forward | 5’-TCT CGG AGA TCC AGC AGA CT-3’ | This study |
| Heat shock protein 27-reverse | 5’-GGA AAT GAA GCC GTG CTC AT-3’ | This study |
| Heat shock protein 72-forward | 5’-AGC GGT ACA AGT CGG AAG AT-3’ | This study |
| Heat shock protein 72-reverse | 5’-TGA TCA CCT CCT GAC ACT TGT-3’ | This study |
| Heat shock protein 73-forward | 5’-GTG CTC ATT CAG GTT TAT GAA GGT-3’ | This study |
| Heat shock protein 73-reverse | 5’-CCT GTG CTC TTA TCC ACA GC-3’ | This study |
| Epidermal growth factor-forward | 5’-ACA GCC CTG AAA TGG ATA GAG A-3’ | This study |
| Epidermal growth factor -reverse | 5’-CTC CCT CTG TCT GTC CAA TAG A-3’ | This study |
| Basic fibroblast growth factor-forward | 5’-GAG TGT GTG CAA ACC GTT ATC T-3’ | This study |
| Basic fibroblast growth factor-reverse | 5’-TGC CAC ATA CCA ACT GGA GTA-3’ | This study |
| Hepatocyte growth factor-forward | 5’-CAA TCC AGA GGT ACG CTA CGA-3’ | This study |
| Hepatocyte growth factor-reverse | 5’-TCC CAA CGC TGA CAA ATC TTG-3’ | This study |
| Transforming growth factor beta-forward | 5’-AGC TCC ACG GAG AAG AAC TG-3’ | This study |
| Transforming growth factor beta -reverse | 5’-AGT GTC TAG GCT CCA GAT GTA G-3’ | This study |
| Cyclooxygenase 2-forward | 5’-CTT CCA AGA CGC CAC TTC AC-3’ | This study |
| Cyclooxygenase 2-reverse | 5’-CTT GGG CAT CCA TTG TGC TA-3’ | This study |
| Nitric oxide synthase 2-forward | 5’-CGA GGC AAA CAC CAC ATT CA-3’ | This study |
| Nitric oxide synthase 2-reverse | 5’-TGC TGC TGA GAG CTT TGT TG-3’ | This study |
| CXCL2-forward | 5’-TGG TCA GGA AGT TTG TCT CAA C-3’ | This study |
| CXCL2-reverse | 5’-TCC GCT AAA GCT ACA GCA GTA-3’ | This study |
| Occludin-forward | 5’-ATC ACT ACT GCG TGG TGG AT-3’ | This study |
| Occludin-reverse | 5’-GGG CTG CTC GTC ATA AAT ACG-3’ | This study |
| Zonula occludin 1-forward | 5’-GCT CCT GGA TTT GGA TTT GGA-3’ | This study |
| Zonula occludin 1-reverse | 5’-ACC ATT GCA ACT CGG TCA TT-3’ | This study |
| Zonula occludin 2-forward | 5’-AGC TCC AGG AAG CAC AGA AT-3’ | This study |
| Zonula occludin 2-reverse | 5’-TCC TCT GGG ATC CTG ATA AAG TC-3’ | This study |
| IL6-forward | 5’-TGT CGA GGC TGT GCA GAT TA-3’ | This study |
| IL6-reverse | 5’-GTG GTG GCT TTG TCT GGA TT-3’ | This study |
| IL8-forward | 5’-CAG AGC CAG GAA GAG ACT AGA A-3’ | This study |
| IL8-reverse | 5’-GGC ATC GAA GTT CTG CAC TTA-3’ | This study |
| IL10-forward | 5’-GGG TTG CCA AGC CTT GTC-3’ | This study |
| IL10-reverse | 5’-CTC CAC GGC CTT GCT CTT-3’ | This study |
| CXCL13-forward | 5’-TGA GGT TCA CAC TGG GAT CTC-3’ | This study |
| CXCL13-reverse | 5’-CCC AGG AGG CCA GAT TTG AA-3’ | This study |
| IL1β-forward | 5’-GGA AGT GAT GGC TAA CAA TGG T-3’ | This study |
| IL1β-reverse | 5’-GGC TTC TCC TTT GCC ACA AT-3’ | This study |
| IFNγ-forward | 5’-TTT CAG CTT TGC GTG ACT TTG-3’ | This study |
| IFNγ-reverse | 5’-CAC TCT CCT CTT TCC AAT TCT TCA-3’ | This study |
| TNFα-forward | 5’-CCC AAG GAC TCA GAT CAT CGT-3’ | This study |
| TNFα-reverse | 5’-TGT CCC TCG GCT TTG ACA T-3’ | This study |
