# Supplementary figures and images for: Characterization of the non-glandular gastric region microbiota in Helicobacter suis-infected versus non-infected pigs identifies a potential role for Fusobacterium gastrosuis in gastric ulceration
Source: Vet Res. 2019 May 24;50:39. doi: 10.1186/s13567-019-0656-9 (PMC6534906; doi:10.1186/s13567-019-0656-9)

| 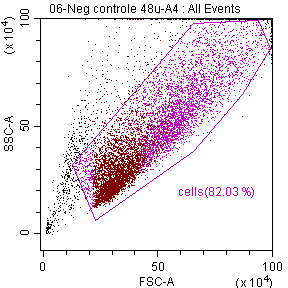  **A**  82.03% | 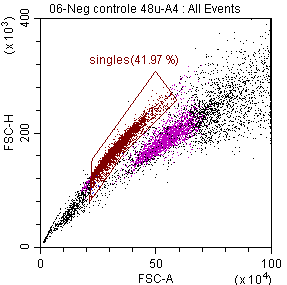  41.97%  **B** |
| --- | --- |
| **C**   |  | **Gain settings** | |  | **Compensation matrix** | | | | --- | --- | --- | --- | --- | --- | --- | |  | **FSC** | 30 |  | **Channel** | **-FITC%** | **-PE%** | |  | **SCC** | 35 |  | FITC |  | 0.00 | |  | **FITC** | 5 |  | PE | 41.07 |  | |  | **PE** | 7 |  |  |  |  |   **E** | 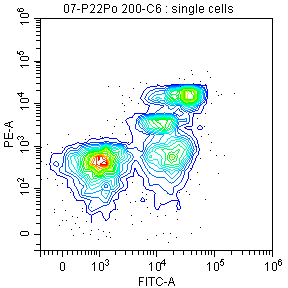  **D** |
| 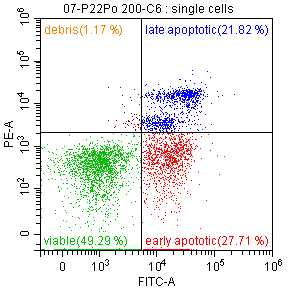 |  |

Supplement: Supplementary file 2 — Additional file 2. Gating strategy of the MKN-7 cell line. (A) FSC-A/SSC-A represents the distribution of cells in the light scatter based on their size and intracellular complexity, respectively. The cells of interest are gated excluding debris. (B) FSC-A/FSC-H allows discrimination between single cells and doublets, single cells are gated. (C) Gain settings and compensation matrix. (D-E) FITC-A/PE-A identifies the selective subpopulations: viable (Annexin-V-FITC negative, PI negative), early apoptotic (Annexin-V-FITC positive, PI negative) and late apoptotic/necrotic (Annexin-V-FITC positive, PI positive) cells. [file 13567_2019_656_MOESM2_ESM.docx]
