# Supplementary material for: Characterization of the non-glandular gastric region microbiota in Helicobacter suis-infected versus non-infected pigs identifies a potential role for Fusobacterium gastrosuis in gastric ulceration
Source: Vet Res. 2019 May 24;50:39. doi: 10.1186/s13567-019-0656-9 (PMC6534906; doi:10.1186/s13567-019-0656-9)
Supplement: Supplementary file 3 — Additional file 3. Gating strategy of the KYSE-450 cell line. (A) FSC-A/SSC-A represents the distribution of cells in the light scatter based on their size and intracellular complexity, respectively. The cells of interest are gated excluding debris. (B) FSC-A/FSC-H allows discrimination between single cells and doublets, single cells are gated. (C) Gain settings and compensation matrix. (D-E) FITC-A/PE-A identifies the selective subpopulations: viable (Annexin-V-FITC negative, PI negative), early apoptotic (Annexin-V-FITC positive, PI negative), late apoptotic/necrotic (Annexin-V-FITC positive, PI positive) and late necrotic (Annexin-V-FITC negative, PI positive) cells. [file 13567_2019_656_MOESM3_ESM.docx]

| 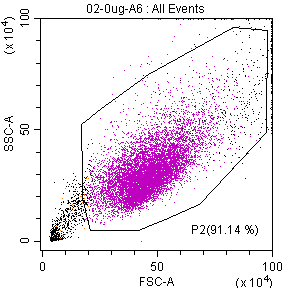  **A**  91.14% | 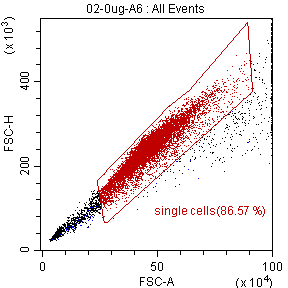  **B**  **D**  86.57% |
| --- | --- |
| **C**   \|  \| **Gain settings** \| \|  \| **Compensation matrix** \| \| \| \| --- \| --- \| --- \| --- \| --- \| --- \| --- \| \|  \| **FSC** \| 50 \|  \| **Channel** \| **-FITC%** \| **-PE%** \| \|  \| **SCC** \| 32 \|  \| FITC \|  \| 0.00 \| \|  \| **FITC** \| 1 \|  \| PE \| 41.07 \|  \| \|  \| **PE** \| 1 \|  \|  \|  \|  \| | 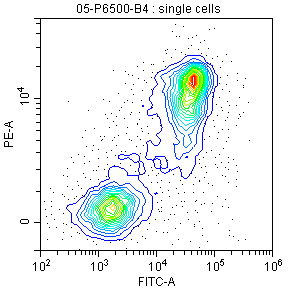 |
| 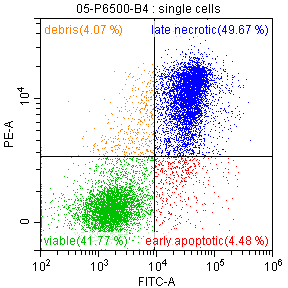  late necrotic (4.07%)  late apoptotic (49.67%)  **E** |  |
