# Supplementary material for: Characterization of the non-glandular gastric region microbiota in Helicobacter suis-infected versus non-infected pigs identifies a potential role for Fusobacterium gastrosuis in gastric ulceration
Source: Vet Res. 2019 May 24;50:39. doi: 10.1186/s13567-019-0656-9 (PMC6534906; doi:10.1186/s13567-019-0656-9)
Supplement: Supplementary file 4 — Additional file 4. Bacterial community compositions present in the Pars oesophagea of each individual pig. The cumulated histograms show the relative abundance of the identified taxa at phylum (A), family (B) and genus (C) level. At family and genus level, taxa with a relative abundance <1% are merged in the category “others”. 1–10 = H. suis-negative pigs, 11–20 = H. suis-positive pigs. The unclassified populations correspond to defined groups of the genus level for which a taxonomical classification assignation to the genus cannot be attributed. These populations are therefore labelled with the first defined superior hierarchical taxonomic level followed by “_unclassified” to prevent confusion. [file 13567_2019_656_MOESM4_ESM.docx]

| **B** |
| --- |
| **C**  100  80  60  40  20  0  100  80  60  40  20  0 |
| 100  80  60  40  20  0 |

**A**
