# Supplementary material for: Characterization of the non-glandular gastric region microbiota in Helicobacter suis-infected versus non-infected pigs identifies a potential role for Fusobacterium gastrosuis in gastric ulceration
Source: Vet Res. 2019 May 24;50:39. doi: 10.1186/s13567-019-0656-9 (PMC6534906; doi:10.1186/s13567-019-0656-9)
Supplement: Supplementary file 5 — Additional file 5. Overview of the bacterial richness (A), diversity (B) and evenness (C) of the Pars oesophagea of H. suis-positive and -negative pigs. The data are represented as scatter plots: each dot represents a pig, the middle line represents the median and the whiskers represent the standard error or the mean. [file 13567_2019_656_MOESM5_ESM.docx]

|   **A** |   **B** |
| --- | --- |
|   **C** |  |
