# Supplementary material for: Characterization of the non-glandular gastric region microbiota in Helicobacter suis-infected versus non-infected pigs identifies a potential role for Fusobacterium gastrosuis in gastric ulceration
Source: Vet Res. 2019 May 24;50:39. doi: 10.1186/s13567-019-0656-9 (PMC6534906; doi:10.1186/s13567-019-0656-9)
Supplement: Supplementary file 6 — Additional file 6. The number of F. gastrosuis bacteria in the different stomach regions of H. suis-positive and -negative 2–3 months old pigs (A), 6–8 months old pigs (B), adult sows (C) and the pigs used for the metagenomics study (D). Data are shown as log10 values of the average number of F. gastrosuis bacteria per mg tissue with standard deviation. Statistical differences were calculated using the non-parametric Kruskal-Wallis H test. *, p < 0.05. [file 13567_2019_656_MOESM6_ESM.docx]

| *□ H. suis - ■ H. suis* +  **A**  **C** | *□ H. suis - ■ H. suis* +  **D**  **B** |
| --- | --- |
| *  *□ H. suis - ■ H. suis* + | *  *  *  *  *□ H. suis - ■ H. suis* + |
