# Supplementary material for: Characterization of the non-glandular gastric region microbiota in Helicobacter suis-infected versus non-infected pigs identifies a potential role for Fusobacterium gastrosuis in gastric ulceration
Source: Vet Res. 2019 May 24;50:39. doi: 10.1186/s13567-019-0656-9 (PMC6534906; doi:10.1186/s13567-019-0656-9)
Supplement: Supplementary file 7 — Additional file 7. The number of F. gastrosuis bacteria in the oral cavity and gastro-intestinal tract of 2–3 months old pigs. Data are shown as log10 values of the average number of F. gastrosuis bacteria per mg tissue with standard deviation. Statistical differences were calculated using the non-parametric Kruskal-Wallis H test. *, p < 0.05; **, p < 0.001 significant differences between the regions. [file 13567_2019_656_MOESM7_ESM.docx]

*

**

**

*

*
