# Supplementary material for: Characterization of the non-glandular gastric region microbiota in Helicobacter suis-infected versus non-infected pigs identifies a potential role for Fusobacterium gastrosuis in gastric ulceration
Source: Vet Res. 2019 May 24;50:39. doi: 10.1186/s13567-019-0656-9 (PMC6534906; doi:10.1186/s13567-019-0656-9)
Supplement: Supplementary file 8 — Additional file 8. The number of F. gastrosuis bacteria in the different stomach regions of 2–3 months old pigs, 6–8 months old pigs and adult sows. Data are shown as log10 values of the average number of F. gastrosuis bacteria per mg tissue with standard deviation. Statistical differences were calculated using the non-parametric Kruskal-Wallis H test. *, p < 0.05; **, p < 0.001 significant differences between the stomach regions. Significant differences between the age groups are indicated with brackets. [file 13567_2019_656_MOESM8_ESM.docx]

**

**

**

**

**

**

*

*

**

**

**

**

**
