# Supplementary material for: Characterization of the non-glandular gastric region microbiota in Helicobacter suis-infected versus non-infected pigs identifies a potential role for Fusobacterium gastrosuis in gastric ulceration
Source: Vet Res. 2019 May 24;50:39. doi: 10.1186/s13567-019-0656-9 (PMC6534906; doi:10.1186/s13567-019-0656-9)
Supplement: Supplementary file 9 — Additional file 9. General overview of gene expression analysis of markers for inflammation and ulceration in the Pars oesophagea of H. suis-negative and -positive pigs. The data are presented as fold changes in gene expression normalized to 3 reference genes and relative to a H. suis-negative control pigs. The fold changes are shown as means with the standard error of the mean. Statistical differences were calculated using the non-parametric Kruskal-Wallis H test. *, p < 0.05; significant differences between the H. suis-positive pigs and -negative pigs. [file 13567_2019_656_MOESM9_ESM.docx]

*

*
