# Supplementary material for: Characterization of the non-glandular gastric region microbiota in Helicobacter suis-infected versus non-infected pigs identifies a potential role for Fusobacterium gastrosuis in gastric ulceration
Source: Vet Res. 2019 May 24;50:39. doi: 10.1186/s13567-019-0656-9 (PMC6534906; doi:10.1186/s13567-019-0656-9)
Supplement: Supplementary file 10 — Additional file 10. Overview of relative fold changes of altered markers for inflammation and ulceration in the Pars oesophagea of H. suis-positive pigs. The data are presented as fold changes in gene expression normalized to 3 reference genes and relative to the H. suis-negative pigs. The fold changes are shown as means with the standard error of the mean. Statistical differences were calculated using the non-parametric Kruskal-Wallis H test. A p-value lower than 0.05 is considered to be significant. [file 13567_2019_656_MOESM10_ESM.docx]

**Additional file 10:** Overview of relative fold changes of altered markers for inflammation and ulceration in the *Pars oesophagea* of *H. suis*-positive pigs.

| **Gene** | **Relative fold change** | ***p*-value** |
| --- | --- | --- |
|  |  |  |
| Claudin 18 | 5.23 ± 1.69 | 0.050 |
| Hsp 72 | 4.06 ± 1.55 | 0.211 |
| IL-8 | 2.62 ± 0.73 | 0.139 |
|  |  |  |
| Claudin 2 | 0.81 ± 0.66 | 0.043 |
| Claudin 3 | 0.46 ± 0.18 | 0.093 |
| CXCL2 | 0.58 ± 0.13 | 0.065 |
