# Supplementary material for: Characterization of the non-glandular gastric region microbiota in Helicobacter suis-infected versus non-infected pigs identifies a potential role for Fusobacterium gastrosuis in gastric ulceration
Source: Vet Res. 2019 May 24;50:39. doi: 10.1186/s13567-019-0656-9 (PMC6534906; doi:10.1186/s13567-019-0656-9)
Supplement: Supplementary file 11 — Additional file 11. Correlation between the number of colonizing F. gastrosuis bacteria per mg gastric tissue and the expression of markers for inflammation and ulceration. The data are presented as a scatter plot: each dot represents the individual data of a pig. The trendline shows the relationship between the relative mRNA expression of a marker for inflammation/ulceration and the log10 values of the number of F. gastrosuis bacteria per mg gastric tissue. y = equation of the trendline. r = Pearson correlation coefficient, calculated using SPSS Statistics 24. A r-value close to 1 indicates a strong, positive correlation, whereas a r-value of −1 indicates a strong, negative correlation. P-values lower than 0.05 are considered to be significant. [file 13567_2019_656_MOESM11_ESM.docx]

|  |  |
| --- | --- |
|  |  |
|  |  |
|  |  |
