# Supplementary material for: Characterization of the non-glandular gastric region microbiota in Helicobacter suis-infected versus non-infected pigs identifies a potential role for Fusobacterium gastrosuis in gastric ulceration
Source: Vet Res. 2019 May 24;50:39. doi: 10.1186/s13567-019-0656-9 (PMC6534906; doi:10.1186/s13567-019-0656-9)
Supplement: Supplementary file 12 — Additional file 12. Percentage of viable, early apoptotic and late apoptotic/necrotic MKN-7 cells after incubation with F. gastrosuis lysate. Data are shown as the average (n = 3) percentages of viable (green), early apoptotic (red) and late apoptotic/necrotic (blue) MKN-7 cells with standard deviation. The cells were incubated for 24 (A), 36 (B) and 48h (C) with 50 µg, 200 µg and 500 µg F. gastrosuis lysate (4 strains, CDW1, 3, 6 and 8) and F. necrophorum subsp. necrophorum (Fnn) as positive control. * Significant differences between the negative control and cells incubated with each bacterial lysate (p < 0.05). [file 13567_2019_656_MOESM12_ESM.docx]

| **A** |
| --- |
| **B**  *  *  *  *  *  *  *  *  *  *  *  *  *  *  *  *  *  *  *  *  *  *  *  *  *  *  *  *  *  *  *  *  *  *  *  *  *  *  *  *  *  *  30  50  70  100  80  60  40  20  0  10  90  30  50  70  100  80  60  40  20  0  10  90 |
| **C**  30  50  70  100  80  60  40  20  0  10  90  *  *  *  *  *  *  *  *  *  *  *  *  *  *  *  *  *  *  *  *  *  *  *  *  *  *  *  *  *  *  *  *  *  *  *  *  *  *  *  *  *  *  *  *  *  *  *  *  *  *  *  *  *  * |
