# Supplementary material for: Characterization of the non-glandular gastric region microbiota in Helicobacter suis-infected versus non-infected pigs identifies a potential role for Fusobacterium gastrosuis in gastric ulceration
Source: Vet Res. 2019 May 24;50:39. doi: 10.1186/s13567-019-0656-9 (PMC6534906; doi:10.1186/s13567-019-0656-9)
Supplement: Supplementary file 13 — Additional file 13. Percentage of viable, early apoptotic and late apoptotic/necrotic MKN-7 cells after incubation with viable F. gastrosuis bacteria. Data are shown as the average (n = 3) percentages of viable (green), early apoptotic (red) and late apoptotic/necrotic (blue) MKN-7 cells with standard deviation. The cells were incubated for 2 (A), 6 (B) and 12h (C) with 5 MOI, 20 MOI and 50 MOI viable F. gastrosuis bacteria (4 strains, CDW1, 6, 6 and 8) and F. necrophorum subsp. necrophorum (Fnn) as positive control. * Significant differences between the negative control and cells incubated with each bacterial strain (p < 0.05). [file 13567_2019_656_MOESM13_ESM.docx]

| **A**  *  *  *  *  *  *  *  *  *  *  *  *  *  *  *  *  *  *  *  *  *  *  *  *  *  *  *  *  *  *  * |
| --- |
| **B**  30  50  70  100  80  60  40  20  0  10  90  30  50  70  100  80  60  40  20  0  10  90  *  *  *  *  *  *  *  *  *  *  *  *  *  *  *  *  *  *  *  *  *  *  *  *  *  *  *  *  *  *  *  *  * |
| **C**  30  50  70  100  80  60  40  20  0  10  90  *  *  *  *  *  *  *  *  *  *  *  *  *  *  *  *  *  *  *  *  *  *  *  *  *  *  *  *  *  *  *  * |
