# Supplementary material for: Characterization of the non-glandular gastric region microbiota in Helicobacter suis-infected versus non-infected pigs identifies a potential role for Fusobacterium gastrosuis in gastric ulceration
Source: Vet Res. 2019 May 24;50:39. doi: 10.1186/s13567-019-0656-9 (PMC6534906; doi:10.1186/s13567-019-0656-9)
Supplement: Supplementary file 14 — Additional file 14. Visualization of MKN-7 cellular morphology using hemacolor staining and detection of early apoptotic and late apoptotic/necrotic cells using flow cytometry. (A-D) Hemacolor staining of MKN-7 cells incubated (A) without F. gastrosuis for 48h and (B-D) with 500 µg F. gastrosuis strain CDW1 incubated for (B) 24 h, (C) 36 h and (D) 48h. Following morphologic features can be seen: plasma membrane blebbing (white arrow), cell swelling (white star), pyknosis (black arrow), cytoplasmic vacuoles (black star). Original magnification x400, scale bar represents 10 µm. (E-L) Representative population plots displaying viable (green, Annexin-V-FITX negative, PI negative), early apoptotic (red, Annexin-V-FITX positive, PI negative), late apoptotic/necrotic (blue, Annexin-V-FITX positive, PI positive) cells of MKN-7 cells incubated (E) without F. gastrosuis lysate for 48 h; (F-H) with 500 µg F. gastrosuis strain CDW1 for (F) 24 h; (G) 36 h and (H) 48 h; (I) without viable F. gastrosuis bacteria for 12h; (J-L) with 50 MOI F. gastrosuis bacteria strain CDW8 for (J) 2h; (K) 6h and (L) 12h. Y-axis: propidium iodide (PE) signal intensity; X-axis: Annexin-V-fluorescein isothiocyanate (FITC) signal intensity. The percentage of population plots is presented in the corresponding gate. [file 13567_2019_656_MOESM14_ESM.docx]

| Negative control | 500 µg *F. gastrosuis* lysate, 24h | 500 µg *F. gastrosuis* lysate, 36h | 500 µg *F. gastrosuis* lysate, 48h |
| --- | --- | --- | --- |
| 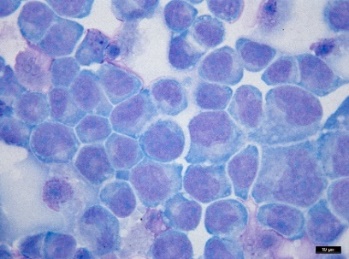 | 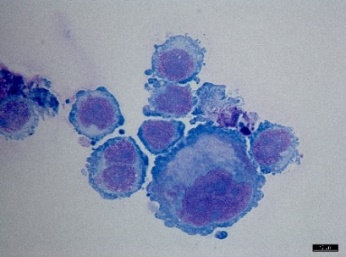 | 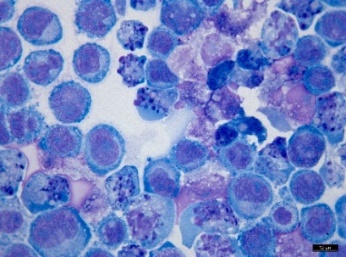 | 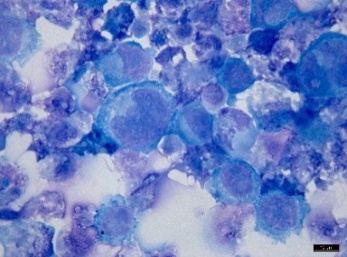 |
|  |  |  |  |
| Negative control | 500 µg *F. gastrosuis* lysate, 24h | 500 µg *F. gastrosuis* lysate, 36h | 500 µg *F. gastrosuis* lysate, 48h |
| 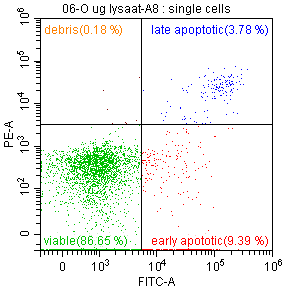 | 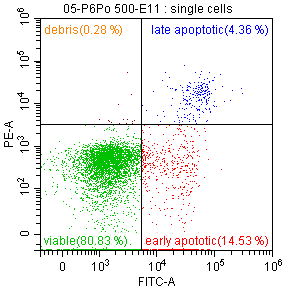 | 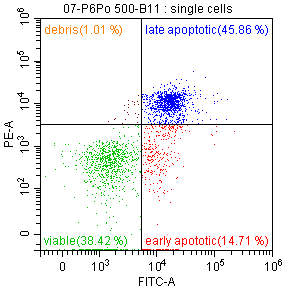 | 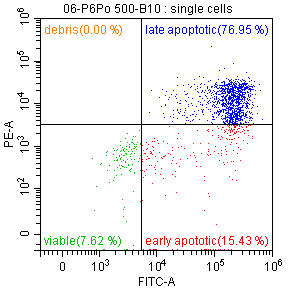 |
|  |  |  |  |
| Negative control | 50 MOI *F. gastrosuis* bacteria, 2h | 50 MOI *F. gastrosuis* bacteria, 6h | 50 MOI *F. gastrosuis* lysate, 12h |
| 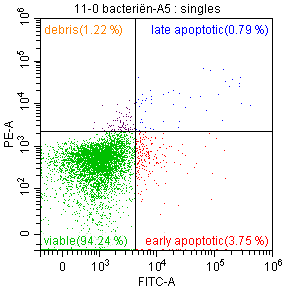 | 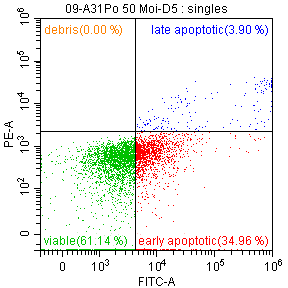 | 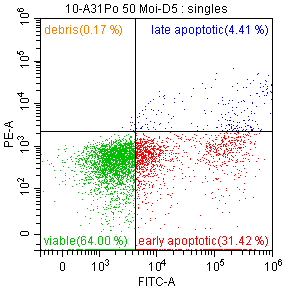 | 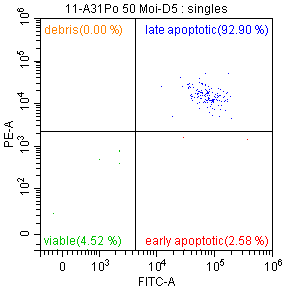 |

**D**

**C**

**B**

**A**

**E**

**F**

**G**

**H**

**I**

**J**

**K**

**L**
