# Supplementary material for: Characterization of the non-glandular gastric region microbiota in Helicobacter suis-infected versus non-infected pigs identifies a potential role for Fusobacterium gastrosuis in gastric ulceration
Source: Vet Res. 2019 May 24;50:39. doi: 10.1186/s13567-019-0656-9 (PMC6534906; doi:10.1186/s13567-019-0656-9)
Supplement: Supplementary file 15 — Additional file 15. Percentage of viable, early apoptotic, late apoptotic/necrotic and late necrotic KYSE-450 cells after incubation with F. gastrosuis lysate. Data are shown as the average (n = 3) percentages of viable (green), early apoptotic (red), late apoptotic/necrotic (blue) and late necrotic (orange) KYSE-450 cells with standard deviation. The cells were incubated for 24 (A), 36 (B), 48 (C) and 72 h (D) with 50 µg, 200 µg and 500 µg F. gastrosuis lysate (4 strains, CDW1, 3,6 and 8) and F. necrophorum subsp. necrophorum (Fnn) as positive control. * Significant differences between the negative control and cells incubated with each bacterial lysate (p < 0.05). [file 13567_2019_656_MOESM15_ESM.docx]

| **A**  % late necrotic  30  50  70  100  80  60  40  20  0  10  90  *  *  *  *  *  *  *  *  *  *  *  *  *  *  *  *  *  *  *  *  *  *  *  *  *  *  *  *  *  *  *  *  *  *  *  *  *  *  *  *  *  *  *  *  *  *  *  *  *  *  *  *  * |
| --- |
| **B**  % late necrotic  30  50  70  100  80  60  40  20  0  10  90  *  *  *  *  *  *  *  *  *  *  *  *  *  *  *  *  *  *  *  *  *  *  *  *  *  *  *  *  *  *  *  *  *  *  *  *  *  *  *  *  *  *  *  *  *  *  *  *  *  *  *  *  *  *  *  *  *  *  * |
| **C**  % late necrotic  30  50  70  100  80  60  40  20  0  10  90  *  *  *  *  *  *  *  *  *  *  *  *  *  *  *  *  *  *  *  *  *  *  *  *  *  *  *  *  *  *  *  *  *  *  *  *  *  *  *  *  *  *  *  *  *  *  *  *  *  *  *  *  *  *  *  *  *  *  * |
| **D**  % late necrotic  30  50  70  100  80  60  40  20  0  10  90 |

*

*

*

*

*

*

*

*

*

*

*

*

*

*

*

*

*

*

*

*

*

*

*

*

*

*

*

*

*

*

*

*

*

*

*

*

*

*

*

*

*

*

*

*

*

*

*

*

*

*

*

*

*

*

*

*

*

*

*

*
