# Supplementary material for: Characterization of the non-glandular gastric region microbiota in Helicobacter suis-infected versus non-infected pigs identifies a potential role for Fusobacterium gastrosuis in gastric ulceration
Source: Vet Res. 2019 May 24;50:39. doi: 10.1186/s13567-019-0656-9 (PMC6534906; doi:10.1186/s13567-019-0656-9)
Supplement: Supplementary file 17 — Additional file 17. Visualization of KYSE-450 cellular morphology using hemacolor staining and detection of early apoptotic and late apoptotic/necrotic cells using flow cytometry. (A-D) Hemacolor staining of KYSE-450 cells incubated (A) without F. gastrosuis for 48 h and (B-D) with 500 µg F. gastrosuis strain CDW1 incubated for (B) 36 h, (C) 48 h and (D) 72 h. Following morphologic features can be seen: plasma membrane blebbing (white arrow), cell swelling (white star), cytoplasmic vacuoles (black star). Original magnification x400, scale bar represents 10 µm. (E-M) Representative population plots displaying viable (green, Annexin-V-FITC negative, PI negative), early apoptotic (red, Annexin-V-FITC positive, PI negative), late apoptotic/necrotic (blue, Annexin-V-FITC positive, PI positive) cells and late necrotic (orange, Annexin-V-FITC negative, PI positive) of KYSE-450 cells incubated (E) without F. gastrosuis lysate for 72 h; (F-I) with 500 µg F. gastrosuis strain CDW1 for (F) 24 h; (G) 36 h; (H) 48 h and (I) 72 h; (J) without viable F. gastrosuis bacteria for 12 h; (K-M) with 50 MOI F. gastrosuis bacteria strain CDW8 for (K) 2 h; (L) 6h and (M) 12 h. Y-axis: propidium iodide (PE) signal intensity; X-axis: Annexin-V-fluorescein isothiocyanate (FITC) signal intensity. The percentage of population plots is presented in the corresponding gate. [file 13567_2019_656_MOESM17_ESM.docx]

**D**

**C**

**B**

**A**

**E**

**F**

**G**

**H**

**I**

**J**

**K**

**L**

late necrotic (1.01%)

late necrotic (0.86%)

late necrotic (5.54%)

late necrotic (2.36%)

late necrotic (4.45%)

late necrotic (0.54%)

late necrotic (0.86%)

late necrotic (1.09%)

late necrotic (7.42%)

0

late apoptotic (2.03%)

late apoptotic (2.53%)

late apoptotic (1.11%)

late apoptotic (10.96%)

late apoptotic (48.72%)

late apoptotic (0.81%)

late apoptotic (1.26%)

late apoptotic (1.90%)

late apoptotic (5.43%)

| Negative control | 500 µg *F. gastrosuis* lysate, 36h | 500 µg *F. gastrosuis* lysate, 48h | 500 µg *F. gastrosuis* lysate, 72h |
| --- | --- | --- | --- |
| 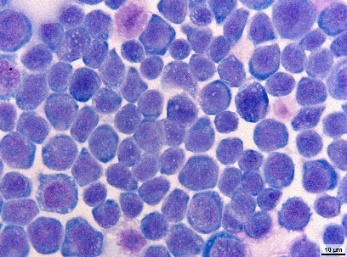 | 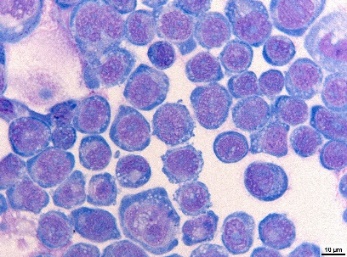 | 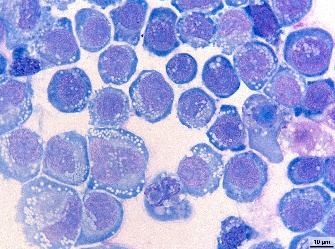 | 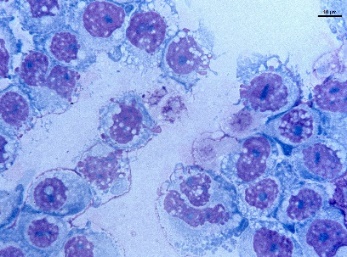 |
| Negative control | 500 µg *F. gastrosuis* lysate, 24h | 500 µg *F. gastrosuis* lysate, 36h | 500 µg *F. gastrosuis* lysate, 48h |
| 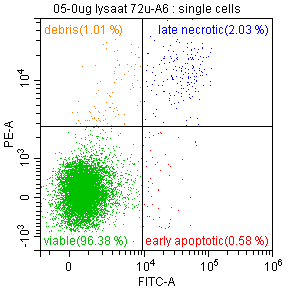 | 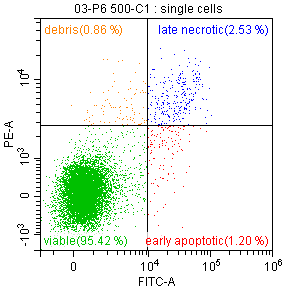 | 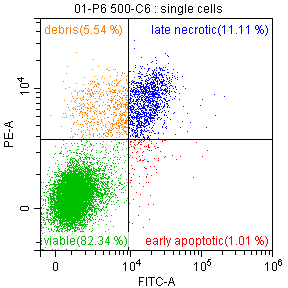 | 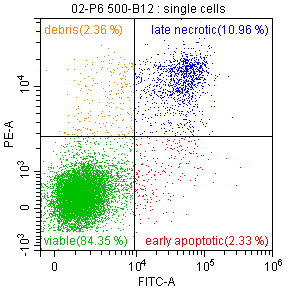 |
| 500 µg *F. gastrosuis* lysate, 72h | Negative control | 50 MOI *F. gastrosuis* bacteria, 2h | 50 MOI *F. gastrosuis* bacteria, 6h |
| 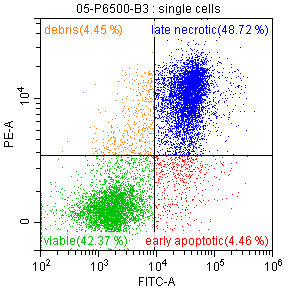 | 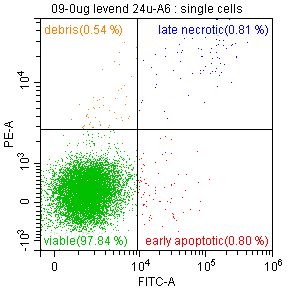 | 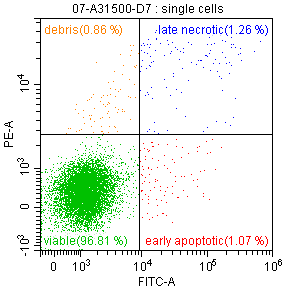 | 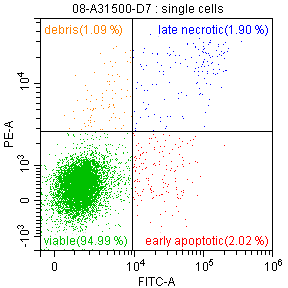 |
|  |  |  |  |
| 50 MOI *F. gastrosuis* lysate, 12h |  |  |  |
| 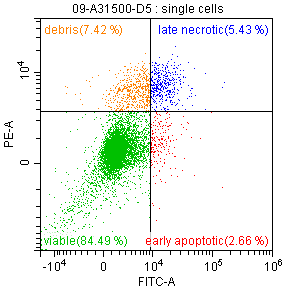  **M** |  |  |  |
